# Selective Neuron Vulnerability in Common and Rare Diseases—Mitochondria in the Focus

**DOI:** 10.3389/fmolb.2021.676187

**Published:** 2021-06-30

**Authors:** Thomas Paß, Rudolf J. Wiesner, David Pla-Martín

**Affiliations:** ^1^ Center for Physiology and Pathophysiology, Institute of Vegetative Physiology, University of Cologne, Cologne, Germany; ^2^ Cologne Excellence Cluster on Cellular Stress Responses in Aging Associated Diseases (CECAD), University of Cologne, Cologne, Germany

**Keywords:** mitochondria, neurodegeneration, selective vulnerability, mitochondrial DNA maintenance, mitochondrial dynamics, quality control, Ca^2+^ homeostasis

## Abstract

Mitochondrial dysfunction is a central feature of neurodegeneration within the central and peripheral nervous system, highlighting a strong dependence on proper mitochondrial function of neurons with especially high energy consumptions. The fitness of mitochondria critically depends on preservation of distinct processes, including the maintenance of their own genome, mitochondrial dynamics, quality control, and Ca^2+^ handling. These processes appear to be differently affected in common neurodegenerative diseases, such as Alzheimer’s and Parkinson’s disease, as well as in rare neurological disorders, including Huntington’s disease, Amyotrophic Lateral Sclerosis and peripheral neuropathies. Strikingly, particular neuron populations of different morphology and function perish in these diseases, suggesting that cell-type specific factors contribute to the vulnerability to distinct mitochondrial defects. Here we review the disruption of mitochondrial processes in common as well as in rare neurological disorders and its impact on selective neurodegeneration. Understanding discrepancies and commonalities regarding mitochondrial dysfunction as well as individual neuronal demands will help to design new targets and to make use of already established treatments in order to improve treatment of these diseases.

## Introduction

Mitochondria are dynamic, double-membrane-surrounded organelles executing a wide range of essential functions within the cell, including ATP production, metabolism of amino acids, lipids and nucleotides, iron-sulfur cluster synthesis, ion homeostasis, and programmed cell death. The pivotal role of mitochondria for cellular survival is highlighted by the variety of diseases that are associated with mitochondrial dysfunction in diverse tissues. Neurons especially depend on proper mitochondrial function due to their extremely high energetic demands, with maintaining resting membrane potentials and firing of action potentials being the largest energy guzzlers (Howarth et al., 2012). Whereas accounting for only 2% of the whole body mass, the brain consumes 20% of the body’s total oxygen amounts during ATP generation (Attwell and Laughlin 2001). 75–80% of the brains’ energy is thereby used up by neurons (Harris et al., 2012; Hyder et al., 2013), and in contrast to other neural cells, mitochondrial OXPHOS carries most of the burden (Belanger et al., 2011; Rangaraju et al., 2014; Zhang et al., 2014). Therefore, neurons are locally supported by astrocytes, providing additional lactate (Weber and Barros 2015). Moreover, neuronal mitochondria serve as dynamic key regulators of intracellular Ca^2+^. In collaboration with the endoplasmic reticulum (ER), mitochondria control somato-dendritic Ca^2+^ levels (Rizzuto et al., 1998; Kornmann et al., 2009; Hirabayashi et al., 2017) and buffer Ca^2+^ at presynaptic terminals as well as axonal boutons, in order to regulate neurotransmission (Billups and Forsythe 2002; Kwon et al., 2016; Marland et al., 2016; de Juan-Sanz et al., 2017; Vaccaro et al., 2017). Maintenance of mitochondrial fitness is therefore of great importance and requires efficient quality control mechanisms (Rugarli and Langer 2012), which are challenging regarding the extended and complex neuron morphology (Misgeld and Schwarz 2017).

Perturbations of mitochondrial functions, whether they are of primary cause or not, are accordingly associated with neuronal death in common as well as rare neurological disorders. Mitochondrial dysfunction becomes apparent by impaired activity of respiratory chain complexes, which eventually impacts oxidative phosphorylation (OXPHOS), and thereby ATP generation. Respiratory chain deficiency is found in patients suffering from Alzheimer’s disease (AD), Parkinson’s disease (PD), Huntington’s disease (HD), Amyotrophic Lateral Sclerosis (ALS) (Golpich et al., 2017) spinocerebellar ataxia (Lax et al., 2012), and other peripheral neuropathies, such as Charcot-Marie-Tooth disease (Rizzo et al., 2016). However, defects of the respiratory chain are only the tip of the iceberg. In general, they are preceded by disruption of distinct other mitochondrial processes, including mitochondrial DNA (mtDNA) maintenance, mitochondrial dynamics, quality control, and Ca^2+^ handling.

Remarkably, neurodegenerative diseases usually show selective vulnerability of diverse neuron populations, even in familial cases with monogenic mutations ubiquitously present in the body: 1) In AD, pyramidal neurons in the entorhinal cortex layer II (ECII) and the hippocampal CA1 (*cornu ammonis* 1) region degenerate first, causing cognitive decline and memory loss (Hyman et al., 1984; Arnold et al., 1991; Gomez-Isla et al., 1996; Fukutani et al., 2000; Bussiere et al., 2003). 2) In PD patients, characteristic motor symptoms are induced by the loss of midbrain dopaminergic neurons in the *substantia nigra pars compacta* (SNc) (Michel et al., 2016; Obeso et al., 2017), whereas 3) in HD, motor impairment is based on the decline of GABAergic medium spiny neurons located in the striatum (Halliday et al., 1998; Vonsattel 2008). 4) Spinal motor neurons innervating fast-twitch muscles selectively perish in ALS and result in muscle atrophy and spasticity (Nijssen et al., 2017). 5) Spinocerebellar ataxia is mainly caused by the loss of cerebellar Purkinje cells (Durr 2010), and lastly, 6) Charcot-Marie-Tooth disease is characterized by the decline of peripheral nerves (Reilly et al., 2010; Saporta et al., 2011).

The selective neurodegeneration associated with disruption of distinct mitochondrial processes, raises the question whether cell-type specific properties contribute to an enhanced susceptibility to mitochondrial defects. Here, we review distinct sources for mitochondrial dysfunction in selected common and rare neurological disorders, discuss their primary role in degeneration of specific neuron types and summarize current and potential approaches against neurodegeneration. Understanding the vulnerability of different neuronal populations to specific mitochondrial impairment will prove crucial to disease-specific development of therapies as well as to usage of established drugs for disease-spanning treatment.

## Mitochondrial Pathways Affected in Neurological Disorders

For a long time, impaired mitochondrial OXPHOS has been the most obvious and exclusive explanation for neuronal death associated with mitochondrial dysfunction. With growing knowledge, however, mitochondria have no longer been solely restricted to energy supply. Today we know that mitochondria form a mobile and interactive network. Disturbances in mobility, fusion and fission are observed in common as well as rare neurological disorders, many of them being even caused by mitochondrial-related gene mutations, and led to a better understanding of pathological mechanisms underlying those diseases.

### Mitochondrial DNA Maintenance and Disorders

Mitochondria contain their own double-stranded DNA, which is densely packed, containing 37 genes in 16.6 kb of its sequence, and present in thousands of copies in neurons. All of the 13 mRNAs encode subunits of the respiratory chain complexes, while 22 tRNAs and two rRNAs are necessary for the mitochondrial translation machinery. Although presenting only the minority of OXPHOS proteins, the 13 mtDNA-encoded subunits are essential since OXPHOS collapses in the absence of mtDNA expression (Larsson et al., 1998; Gustafsson et al., 2016). Regarding that the mitochondrial proteome consists of ∼1.200 different proteins (Sickmann et al., 2003; Foster et al., 2006), the vast majority of mitochondrial proteins are nuclear-encoded and imported into mitochondria while being translated. Consequently, transcription, translation, maintenance, and replication of mtDNA are regulated by hundreds of nuclear-encoded and imported proteins (Gustafsson et al., 2016). Therefore, inherited mutations of mtDNA but also nuclear genes encoding respiratory chain subunits and proteins regulating mtDNA maintenance, respectively, are causing mtDNA diseases with a wide range of clinical manifestations (Schapira 2012). Symptoms of mtDNA diseases that are related to mutations in the mtDNA only arise when the copy number of mutated molecules surpasses a certain threshold (Picard et al., 2016). The presence of both wild type and mutant mtDNA molecules is known as mtDNA heteroplasmy.

**FIGURE 1:**
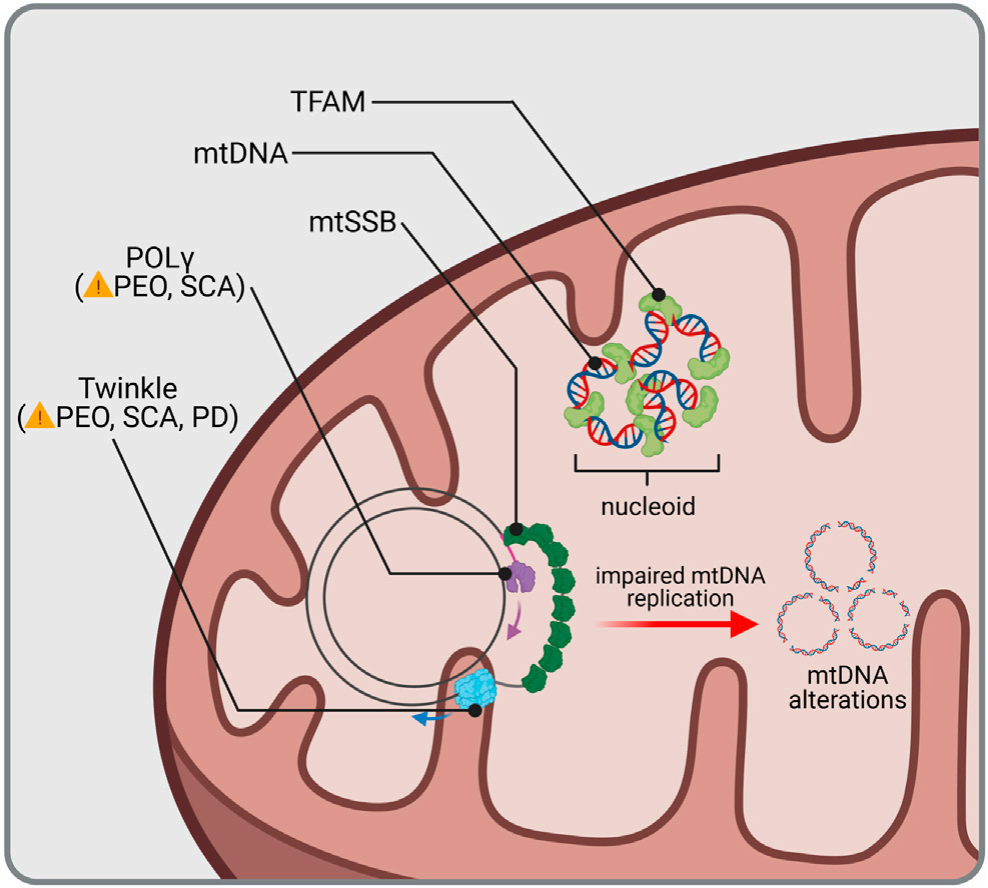
mtDNA homeostasis and related diseases. mtDNA is protected and condensed by TFAM, building an organized DNA-protein complex known as nucleoid. Replication of mtDNA requires coordination of the mitochondrial helicase Twinkle and DNA polymerase γ (POLγ). Mutations in genes encoding for Twinkle and POLγ lead to Progressive External Ophthalmoplegia (PEO), Spinocerebellar Ataxia (SCA) and Parkinsonism (PD). Impaired mtDNA replication is accompanied with mtDNA alterations, such as loss of wild type mtDNA or accumulation of mtDNA deletions, which have not only been found in rare or common diseases but also during aging.

Transcription of mtDNA is initiated by TFAM (mitochondrial transcription factor A), which binds to mitochondrial promoters (Ngo et al., 2011; Shi et al., 2012) and enables recruitment of the mitochondrial RNA polymerase (Yakubovskaya et al., 2014). Simultaneously, TFAM is also responsible for mtDNA maintenance since it entirely coats mtDNA molecules and thus mediates the formation of nucleo-protein structures called nucleoids (Figure 1) (Kaufman et al., 2007; Kukat et al., 2015). In humans, mutation of *TFAM* causes severe mitochondrial depletion syndrome, whereas in mice, the whole body knockout (KO) displayed embryonic lethality due to severe mtDNA depletion (Table 1; Stiles et al., 2016). Interestingly, cell type-specific KO of *Tfam* in dopaminergic neurons mimics the key features of PD pathology in mice. These so called “MitoPark” animals showed rapid and selective loss of dopaminergic neurons in the SNc due to mtDNA depletion, which was accompanied by progressive motor impairment (Ekstrand et al., 2007). Accordingly, low protein levels of TFAM together with reduced mtDNA copy number and complex I deficiency have been detected in SNc dopamine neurons from idiopathic PD patients (Grunewald et al., 2016), suggesting mtDNA depletion to be a critical factor for selective neurodegeneration following mitochondrial dysfunction in PD. Reduced TFAM levels and/or mtDNA copy number have been measured in affected regions of the nervous system from patients of other neurodegenerative diseases as well, including AD (Coskun et al., 2004; Coskun et al., 2012; Rice et al., 2014; Wei et al., 2017), ALS (Keeney and Bennett 2010; Thau et al., 2012; Ladd et al., 2017) and HD (Kim et al., 2010). The vital importance of reduced mtDNA copy number in these disorders is however still disputed. Recently, increased mtDNA levels within the cytosol have been associated with neurodegeneration (Jauhari et al., 2021). Nevertheless, the release of mtDNA, which has been linked to inflammation, is likely to be a consequence of mitochondrial dysfunction in the first place (Sprenger et al., 2021), questioning its potential as a therapeutic target in common and rare neurological disorders.

**TABLE 1:** Proteins related to disturbed mtDNA maintenance.

Protein	Associated disease	Result of malfunction	References
POLγ	Progressive External Ophtalmoplegia (PEO), Spinocerebellar Ataxia, Parkinson’s disease (PD)	mtDNA depletion, accumulation of mtDNA deletions	Lax et al. (2012), Copeland (2014), Chrysostomou et al. (2016)
TFAM	Mitochondrial depletion syndrome	mtDNA depletion	Stiles et al. (2016)
TWINKLE	Progressive External Ophtalmoplegia (PEO), Spinocerebellar Ataxia, Parkinson’s disease (PD)	mtDNA depletion, accumulation of mtDNA deletions	Suomalainen et al. (1997), Lonnqvist et al. (2009), Copeland (2014), Breen et al. (2020)

Besides mtDNA depletion, mitochondrial dysfunction in PD is further linked to the presence of deleted mtDNA molecules. In healthy aged individuals (Kraytsberg et al., 2006) but especially in patients with idiopathic PD (Bender et al., 2006), SNc dopaminergic neurons accumulate high loads of mtDNA deletions which are accompanied with respiratory chain deficiency. Accumulation of deletions is driven by catecholamine metabolism (Neuhaus et al., 2014; Neuhaus et al., 2017), explaining why especially dopamine-expressing neurons are hotspots for such alterations in the mtDNA. Importantly, the high deletion load replaces populations of wild-type mtDNA in PD patients, whereas during normal aging, a sufficient pool of wild-type mtDNA can be maintained by upregulation of the total mtDNA copy number (Dolle et al., 2016). The mechanism for the formation of deleted mtDNA molecules is still not fully understood, however, it is suggested that deletions are generated by misrepaired double strand breaks upon mtDNA damage (Krishnan et al., 2008), followed by nuclease activity preceding re-ligation and during incorrect mtDNA replication (Reeve et al., 2008).

Replication of mtDNA is mediated by DNA polymerase-γ (POLγ). Together with the replicative mtDNA helicase TWINKLE and the mitochondrial single-stranded DNA-binding protein (mtSSB), it forms the mitochondrial replisome, which is able to replicate both mtDNA strands in a continuous manner (Figure 1; Gustafsson et al., 2016). Multiple mutations in *POLG* as well as *TWNK* have been identified and associated with a wide range of diseases usually showing neuromuscular defects, such as progressive external ophthalmoplegia, as a consequence of mtDNA depletion or accumulation of mtDNA deletions (Table 1; Copeland 2014). Ataxia is a prominent clinical feature among mitochondrial diseases related to mutated *POLG* (Van Goethem et al., 2004; Hakonen et al., 2005). Whereas it was first reported to be mostly of sensory nature with no or only mild cerebellar atrophy (Synofzik et al., 2012), Turnbull and colleagues showed that degeneration of Purkinje cells following mitochondrial dysfunction was most pronounced in patients with *POLG* mutations (Lax et al., 2012). In line with this, mtDNA heteroplasmy associated with complex I deficiency was found in cerebellar Purkinje cells of mitochondrial disease patients suffering from ataxia, including patients with *POLG* mutations (Chrysostomou et al., 2016), supporting a cerebellar involvement in ataxias. Patients harboring *TWNK* mutations are presenting with spinocerebellar ataxias (Hudson et al., 2005; Hakonen et al., 2007; Lonnqvist et al., 2009). Correspondingly, respiratory chain-deficient Purkinje cells were detected in mice expressing mutant *Twinkle* (Tyynismaa et al., 2005). Insights of case studies thereby point to special vulnerability of cerebellar Purkinje cells to mtDNA alterations due to impaired replication.

In line with the high load of mtDNA deletions in SNc dopaminergic neurons of idiopathic PD patients, parkinsonian features were additionally seen in some patients harboring mutations of *POLG* (Luoma et al., 2004; Hudson et al., 2007) and *TWNK* (Suomalainen et al., 1997; Baloh et al., 2007; Kiferle et al., 2013). Moreover, mtDNA deletions of idiopathic PD patients were very similar to those found in *POLG* patients (Reeve et al., 2008). Interestingly, extensive study of *POLG* cases indeed revealed severe nigrostriatal degeneration, but lacking characteristic motor symptoms for PD (Tzoulis et al., 2013). The same was true for patients with *TWNK* mutations (Palin et al., 2013), whereas a case of familial parkinsonism with heterozygous mutation in *TWNK* was recently reported (Breen et al., 2020). Taken together, these data indicate that mtDNA maintenance plays a decisive role for survival of SNc dopaminergic neurons and PD pathogenesis.

### Mitochondrial Morphology, Fission and Fusion

From isolated organelles being fixed at a certain place within the cell, the image of mitochondria has changed to an interconnected reticulum, which is continuously separating and fusing. Mitochondrial fission and fusion are dynamic processes which are influenced by the cell’s metabolic needs. Whereas fission generates new mitochondrial particles and can contribute to removal of defective compartments at the same time, fusion is crucial to distribute the load upon increased metabolic demands and mitochondrial damage, respectively (Youle and van der Bliek 2012), leading to constant rejuvenation of the mitochondrial pool. In contrast to their hosting neurons, proteins only last for weeks or even days (Goldberg 2003). In addition, the majority of mitochondrial proteins are encoded in the nucleus (Calvo et al., 2016). Together with the fact that intact mitochondria are substantially transported anterogradely along the axon (Lin et al., 2017), mitochondrial biogenesis is thought to mainly occur in the Soma. Considering mitochondrial transport velocity along the axon (∼0.5 μm/s) and the lifetime of mitochondrial proteins, rejuvenation of those mitochondrial populations, which reside in large distance from their point of origin, is of great importance for neuronal functionality (Misgeld and Schwarz, 2017). In support of this, a recent study suggests the fitness of mitochondria to also depend on its distance from the cell body (Baranov et al., 2019).

**FIGURE 2:**
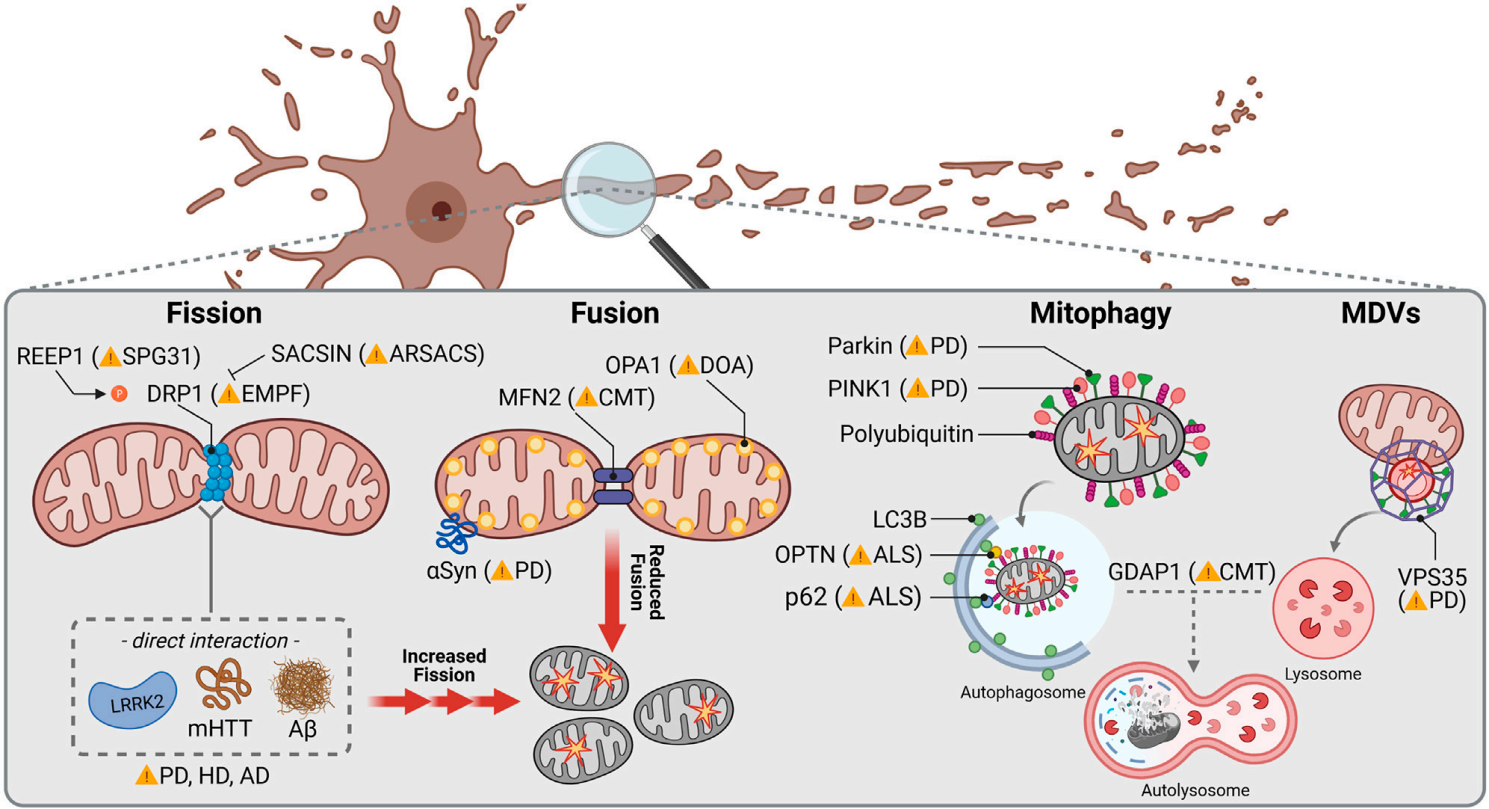
Mitochondrial dynamics in common and rare neurodegenerative diseases. Mitochondrial fission is orchestrated by DRP1, which mutations have been linked to autosomal dominant encephalopathy and neonatal lethality caused by defective mitochondrial and peroxisomal fission. Mutations in REEP1 are associated with autosomal dominant Spastic Paraplegia Type 31 (SPG31) due to hyperphosphorylation of DRP1 causing increased mitochondrial fission. In contrast, *SACSIN* mutations lead to autosomal recessive Spastic Ataxia of Charlevoix-Saguenay (ARSACS) underlying reduced DRP1 recruitment to mitochondrial membranes and decreased fission. PD, HD and AD associated proteins LRKK2, mHTT and Aβ, respectively, have been reported to directly interact with DRP1 stimulating mitochondrial fission. Mitochondrial fusion is regulated by the OMM protein MFN2 and the inner mitochondrial membrane protein OPA1, whose mutations cause autosomal dominant Charcot-Marie-Tooth disease (CMT) and Optic atrophy (DOA), respectively. αSyn interacts with TOM20 which is accompanied with a lower mitochondrial fusion rate. Specific removal of mitochondria through mitophagy in neurons is controlled by PINK1 and Parkin, mutations of both being related to early juvenile recessive PD. PINK1 signalizes depolarized mitochondria by inducing recruitment of Parkin and starting a signaling cascade to remove defective mitochondria through autophagosome-lysosome degradation. Other receptors linked to recessive forms of ALS are the mitophagy specific adaptor protein OPTN and the general autophagy adaptor p62. CMT-related GDAP1 mediates contacts between mitochondria and lysosomes and is hence thought to be involved in mitochondrial removal as well. A higher specialized pathway to eliminate mitochondrial fragments is known to pursue the mitochondrial-derived vesicles (MDVs) trail, where VPS35, linked to dominant forms of PD, triggers the force to generate vesicles which eventually fuse with lysosomes.

Mitochondrial fission is initiated by tubules of the ER constricting a mitochondrial segment, followed by recruitment of the GTPase DRP1 (dynamin-related protein 1), which completes separation (Figure 2) (Friedman et al., 2011). Mutations in *DNML1*, which encodes DRP1, have been associated with rare cases of neonatal encephalopathy and epilepsy (Table 2; Bitoun et al., 2007; Fahrner et al., 2016; Vanstone et al., 2016). Remarkably, other mutations in genes encoding for regulators of DRP1 function have been linked to neurodegenerative disorders: Mutations in *SACSIN* lead to the development of Spastic Ataxia of Charlevoix Saguenay (ARSACS) due to decreased recruitment of DRP1 to mitochondria (Bradshaw et al., 2016). Furthermore, mutations in *REEP1* causing Hereditary Spastic Paraplegia 31 (SPG31) induce hyperphosphorylation of DRP1, hence inhibiting its activity (Lavie et al., 2017).

**TABLE 2:** Proteins related to impaired mitochondrial dynamics.

Protein	Associated disease	Result of malfunction	References
**Mitochondrial fission**			
Aβ	Alzheimer’s disease (AD)	↑ DRP1 protein levels	Cho et al. (2009), Wang et al. (2009)
		↑ mitochondrial fission	Manczak et al. (2011)
DRP1	Neonatal encephalopathy, Epilepsy	↓ mitochondrial fission	Bitoun et al. (2007), Fahrner et al. (2016), Vanstone et al. (2016)
HTT	Huntington’s disease (HD)	↑ DRP1 protein levels	Kim et al. (2010)
		↑ *DRP1* and *FIS1* mRNA levels	Shirendeb et al. (2011)
		↑ mitochondrial fission	Song et al. (2011)
LRRK2	Parkinson’s disease (PD)	↑ mitochondrial fission	Wang et al. (2012), Su and Qi (2013), Smith et al. (2016), Perez-Carrion et al. (2018)
REEP1	Spastic Paraplegia Type 31 (SPG31)	Hyperphosphorylation of DRP1 (↓ mitochondrial fission)	Lavie et al. (2017)
SACSIN	Spastic Ataxia of Charlevoix-Saguenay (ARSACS)	Reduced DRP1 recruitment to mitochondria	Bradshaw et al. (2016)
SOD-1, TDP-43	Amyotrophic Lateral Sclerosis (ALS)	↑ DRP1 and FIS1 protein levels (↑ mitochondrial fission)	Ferri et al. (2010), Xu et al. (2010), Liu et al. (2013), Onesto et al. (2016)
VPS35	Parkinson’s disease (PD)	Increased turnover of DPR1 (↑ mitochondrial fission)	Wang et al. (2016)
**Mitochondrial fusion**			
Aβ	Alzheimer’s disease (AD)	↑ MFN1, MFN2 and OPA1 protein levels	Cho et al. (2009), Wang et al. (2009)
AFG3L2	Spinocerebellar Ataxia	OMA1 activation followed by OPA1 cleavage (↓ mitochondrial fusion)	Ehses et al. (2009), Head et al. (2009)
α-Syn	Parkinson’s disease (PD)	↓ mitochondrial fusion	Devoto et al. (2017)
ATXN3	Spinocerebellar ataxia Type 3	↓ MFN1 and MFN2 protein levels (↓ mitochondrial fusion)	Hsu et al. (2017)
HTT	Huntington’s disease (HD)	↓ MFN1 protein levels	Kim et al. (2010)
		↓ *MFN1*, *MFN2* and *OPA1* mRNA levels	Shirendeb et al. (2011)
LRRK2	Parkinson’s disease (PD)	↓ OPA1 protein levels	Stafa et al. (2014)
MFN2	Charcot-Marie-Tooth disease type 2A (CMT2A)	↓ mitochondrial fusion	Chen et al. (2007), Chen et al. (2010)
OPA1	Optic atrophy	↓ mitochondrial fusion	Amati-Bonneau et al. (2005), Zanna et al. (2008)
SOD-1, TDP-43	Amyotrophic Lateral Sclerosis (ALS)	↓ MFN1 and OPA1 protein levels (↓ mitochondrial fusion)	Ferri et al. (2010), Xu et al. (2010), Liu et al. (2013), Onesto et al. (2016)
VPS35	Parkinson’s disease (PD)	↓ MFN2 protein levels upon MUL1 degradation (↓ mitochondrial fusion)	Tang et al. (2015)
**Mitochondrial transport**			
Aβ	Alzheimer’s disease (AD)	↓ mitochondrial transport	Manczak et al. (2011)
HTT	Huntington’s disease (HD)	↓ mitochondrial transport	Orr et al. (2008), Tian et al. (2014)
LRRK2	Parkinson’s disease (PD)	Accumulation of MIRO1 (↓ mitochondrial transport)	Hsieh et al. (2016)
MFN2	Charcot-Marie-Tooth disease type 2A (CMT2A)	Impaired interaction with MIRO1 (↓ mitochondrial transport)	Detmer et al. (2008), Misko et al. (2010)
Tau	Alzheimer’s disease (AD)	↓ axonal transport	Combs et al. (2019)
**Mitochondrial degradation**			
Aβ	Alzheimer’s disease (AD)	Delayed removal of damaged mitochondria	Shaltouki et al. (2018)
GDAP1	Charcot-Marie-Tooth disease type 4A (CMT4A)	Decreased lysosome function	Cantarero et al. (2020)
LRRK2	Parkinson’s disease (PD)	Delayed removal of damaged mitochondria	Hsieh et al. (2016)
OPTN	Amyotrophic Lateral Sclerosis (ALS)	Accumulation of damaged mitochondria	Wong and Holzbaur (2014), Lazarou et al. (2015), Evans and Holzbaur (2019)
p62	Amyotrophic Lateral Sclerosis (ALS)	Impaired LC3 recognition	Goode et al. (2016), Nozaki et al. (2021)
		↓ autophagy	
Parkin	Parkinson’s disease (PD)	Impaired mitophagy in primary neuronal cells and their axons	Grenier et al. (2014), Ashrafi et al. (2014)
		Degeneration of SNc dopaminergic neurons with impaired mtDNA replication	Pickrell et al. (2015)
PINK1	Parkinson’s disease (PD)	Decreased mitochondrial membrane potential	Amo et al. (2014)
VPS35	Parkinson’s disease (PD)	Delayed removal of damaged mitochondria	Hanss et al. (2020)

Mitochondrial fusion requires merging of the outer mitochondrial membrane (OMM), mediated by mitofusins (MFN1/2), as well as coupling of the inner mitochondrial membrane through OPA1 (optic atrophy 1) (Chen et al., 2003; Eura et al., 2003). While *OPA1* mutations are associated with optic atrophy (Amati-Bonneau et al., 2005; Zanna et al., 2008), mutations in *MFN2* are found in patients with Charcot-Marie-Tooth disease axonal form type 2A (CMT2A), an autosomal dominant form of Charcot-Marie-Tooth (Table 2; Zuchner et al., 2006). This subtype is characterized by peripheral neuropathy primarily affecting motor neurons (Verhoeven et al., 2006; Feely et al., 2011). According to the absence of OPA1, loss of MFN2 has been shown to cause fragmentation of the mitochondrial network in cultured embryonic fibroblasts and membrane potential breakdown in a subpopulation of the arising fragmented mitochondria (Chen et al., 2007; Chen et al., 2010). Besides fusion, MFN2 is necessary for transport of axonal mitochondria through interaction with MIRO1 (mitochondrial Rho GTPase 1) (Misko et al., 2010). MIRO1 is an OMM protein anchoring the microtubule motors kinesin and dynein to mitochondria (Melkov and Abdu 2018). Accordingly, mitochondria are inadequately distributed along axons in a transgenic mouse model of CMT2A carrying a pathogenic *Mfn2* mutation (Detmer et al., 2008).

In line with the strong dependence of SNc dopaminergic neurons on proper mitochondrial functionality, KO of *Mfn2* in dopaminergic neurons leads to severe motor symptoms in mice due to early loss of axonal projections (Lee et al., 2012; Pham et al., 2012). PINK1 and Parkin, whose encoding genes are associated with recessive PD when mutated, have been shown to regulate MFN1 and MFN2 levels (Yang et al., 2008; Tanaka et al., 2010; Glauser et al., 2011). Impaired mitochondrial fusion is suggested to contribute to progressive degeneration of SNc dopaminergic neurons in PD. Indeed, investigation of *post mortem* brain samples of idiopathic PD patients revealed decreased levels of the short form of OPA1 (Zilocchi et al., 2018).

Furthermore, mutations of *LRRK2* causing autosomal dominant PD (Zimprich et al., 2004), are linked to altered mitochondrial morphology (Yue et al., 2015), including elongated mitochondria (Mortiboys et al., 2010). However, most studies reported mitochondrial fragmentation, probably induced by decreased levels of the short form of OPA1 (Stafa et al., 2014) as well as direct interaction of mutant *LRRK2* with DRP1, promoting mitochondrial fission (Figure 2; Wang et al., 2012; Su and Qi 2013; Smith et al., 2016; Perez Carrion et al., 2018). Mutant *LRRK2* further affects mitochondrial trafficking. Whereas under physiological conditions LRRK2 is thought to promote MIRO1 removal, mutant *LRRK2* disrupts this function, resulting in accumulation of the anchoring protein and hence in delay of proper mitochondrial removal. According to similarly enhanced protein levels of MIRO1 in fibroblasts from idiopathic PD patients, mitochondrial trafficking is thought to be generally altered in PD (Hsieh et al., 2016). Noteworthy, mitochondrial dynamics in PD can be affected by α-Syn. Aggregates of α-Syn are the main component of intracellular Lewy bodies, the cellular hallmark for many idiopathic PD cases (Spillantini et al., 1997) as well as familial forms showing mutations in the α-Syn encoding gene *SNCA* (Polymeropoulos et al., 1997). α-Syn, and especially its aggregated form (Wang et al., 2019), is able to bind to the OMM (Di Maio et al., 2016; Hu et al., 2019). Interaction with α-Syn has been shown to decrease mitochondrial fusion rate (Figure 2; Devoto et al., 2017). Moreover, transgenic mice overexpressing α-Syn revealed reduced levels of MFN1 and MFN2, which was accompanied by shortened mitochondria (Xie and Chung 2012), whereas knockdown of α-Syn resulted in mitochondrial elongation (Kamp et al., 2010).

Morphological alterations of mitochondria were one of the first observations made in affected neurons of ALS patients (Atsumi 1981; Sasaki and Iwata 2007). Like in CMT2A, long motor neurons primarily perish. In contrast, only 10% of ALS cases have a familial background, including the most frequently mutated genes *SOD1* (superoxide dismutase 1), *TDP-43* (TAR DNA binding protein) and *FUS* (fused in sarcoma). The majority of cases, however, remain idiopathic (Zou et al., 2017). *In vitro* as well as *in vivo* models of ALS, which have been established in order to investigate the consequences upon expression of mutant *SOD1*, *TDP-43* and *FUS*, respectively, revealed both aggregation and fragmentation of mitochondria (Dal Canto and Gurney 1994; Higgins et al., 2003; De Vos et al., 2007; Vande Velde et al., 2011; Hong et al., 2012; Wang W et al., 2013; Magrane et al., 2014), suggesting impaired mitochondrial dynamics to be a disease-contributing factor. According to this, many groups reported an imbalance between levels of fission and fusion protein: transgenic mouse as well as cell culture models expressing *SOD1 G93A* and mutant *TDP-43*, respectively, showed decreased levels of fusion-mediating proteins, such as MFN1 and OPA1, with simultaneously increased fission-mediating proteins, like DRP1 and FIS1 (Ferri et al., 2010; Xu et al., 2010; Liu et al., 2013; Onesto et al., 2016). Protein levels thereby point to a condition promoting fission, explaining shortened and fragmented mitochondria.

In HD, which is known to be caused by a CAG repeat expansion in the huntingtin gene (*HTT*) generating an expanded polyglutamine stretch in the HTT protein (MacDonald et al., 1993), analysis of striatal lysates from patients indicates a shift to mitochondrial fission, too. Similar to observations made in ALS, increased DRP1 and simultaneously reduced MFN1 levels were measured (Kim et al., 2010), which was further supported by mRNA expression levels showing upregulation of *DRP1* and *FIS1* towards downregulation of *MFN1*, *MFN2* and *OPA1* (Shirendeb et al., 2011). Correspondingly, fragmented mitochondria have been detected in striatal cells from mutant HTT (mHTT) transgenic mice (Napoli et al., 2013). In particular, mHTT abnormally binds DRP1 (Figure 2), which led to mitochondrial fragmentation in transgenic rodent HD models as well as in fibroblasts from HD patients (Song et al., 2011). Simultaneously, mHTT blocks mitochondrial transport (Orr et al., 2008; Tian et al., 2014), especially when it contains polyglutamine repeats (Chang et al., 2006).

Due to their clinical manifestation in mitochondrial DNA diseases (Lax et al., 2012), cerebellar ataxias have been investigated for abnormalities in mitochondrial morphology as well. Interestingly, both fragmentation and elongation of mitochondria have been observed in related model organisms. Mutant *ATXN3*, which is linked to spinocerebellar ataxia type 3 (Kawaguchi et al., 1994), caused mitochondrial fission in neuroblastoma cells and transgenic mice, with decreased protein levels of MFN1 and MFN2 (Hsu et al., 2017). Based on a study expressing mutant *ppp2r2b* in *Drosophila melanogaster*, mitochondrial fragmentation induced by fission is also thought to occur in spinocerebellar ataxia type 12 (Wang et al., 2011). Another type of spinocerebellar ataxia is referred to mutations in *AFG3L2*. AFG3L2 is a subunit of *m*-AAA proteases, whose loss affects mitochondrial protein synthesis and respiration (Patron et al., 2018). The absence of AFG3L2 in Purkinje cells triggers mitochondrial fragmentation as well as altered distribution of mitochondria in the dendritic tree (Almajan et al., 2012). Fragmented mitochondria and defective mitochondrial trafficking were further observed in murine cortical neurons following AFG3L2 depletion (Kondadi et al., 2014). In particular, it is suggested that the inner membrane peptidase OMA1 is activated by absence of the *m*-AAA protease (Ehses et al., 2009), triggering OPA1 cleavage (Head et al., 2009) and thereby facilitating mitochondrial fission (Anand et al., 2014). Mice expressing mutant *ATXN7*, which is linked to spinocerebellar ataxia type 7, revealed mitochondrial network fragmentation in Purkinje cells. Counterintuitively, enlarged mitochondria have been identified by ultrastructural analysis (Ward et al., 2019). In addition, brain-specific loss of DRP1 causes degeneration of Purkinje cells with simultaneously oversized mitochondria failing to spread into neuronal projections (Delettre et al., 2000; Ishihara et al., 2009). These observations are supported by an animal model of spastic ataxia of Charlevoix-Saguenay, in which mice do not express the DRP1-binding protein sacsin: animals showed similar features to mice lacking DRP1, including hyperfused mitochondria and loss of Purkinje cells (Girard et al., 2012), rendering proper mitochondrial fission of critical importance for Purkinje cell survival.

Investigation of patient-derived fibroblasts and *in vitro* models exposed to high Aβ levels pointed to mitochondrial fragmentation and accumulation of dysfunctional mitochondria in AD (DuBoff et al., 2013; Nixon 2013). Some studies could correspondingly demonstrate increased DRP1 as well as decreased MFN1, MFN2 and OPA1 protein levels in brains of AD patients as well as mice (Cho et al., 2009; Wang et al., 2009), whereas others showed conflicting results (Wang et al., 2008). Aβ aggregates could play a critical role for the observed disturbances in mitochondrial dynamics: impaired transport and fragmentation of mitochondria were observed upon overexpression of the Aβ precursor protein in primary neurons, which was connected to a direct interaction of Aβ and DRP1 (Figure 2; Manczak et al., 2011). Interestingly, inhibition of mitochondrial fission could ameliorate aberrant Aβ accumulation in transgenic mice, which was associated with prevention of cognitive deficits (Wang et al., 2017). This indicates that impaired mitochondrial dynamics is not only a consequence of aberrant Aβ aggregates, but could affect plaque formation and might hence be a critical factor for AD pathogenesis. Besides Aβ, pTau is thought to interfere with mitochondrial trafficking. Usually binding and stabilizing microtubules, Tau detaches from microtubules in its hyperphosphorylated form causing microtubule destabilization (Alonso et al., 1994; LeBoeuf et al., 2008). Although the exact mechanism how pTau affects mitochondrial function still remains unclear, it is most likely, that general impairment of axonal transport due to pTau (Combs et al., 2019) also hits mitochondrial movement. In addition, Aβ also affects the processing of pre-proteins imported from the cytosol and thereby mitochondrial proteostasis (Mossmann et al., 2014), leading to instability of OXPHOS complexes, impaired oxygen consumption and reduced mitochondrial membrane potential.

### Mitophagy

Besides constant rejuvenation *via* fission and fusion, efficient quality control is indispensable in order to maintain a healthy mitochondrial pool in somatic and especially axonal compartments. Autophagy functions as a neuronal safeguard and removes large compartments of the mitochondrial network in a controlled manner, which is replaced by ongoing biogenesis. This so called mitophagy is initiated by the mitochondrial serine/threonine kinase PINK1 (PTEN-induced putative kinase 1). Upon mitochondrial damage, PINK1 is no longer cleaved by mitochondrial proteases, such as PARL (Jin et al., 2010; Deas et al., 2011), which is usually followed by its import *via* the TOM (translocase of the outer membrane) (Lazarou et al., 2012) and TIM (translocase of the inner membrane) complexes (Sekine and Youle 2018). This leads to the accumulation of PINK1 on the OMM (Lin and Kang 2008; Zhou et al., 2008). PINK1 hence phosphorylates ubiquitin chains and activates the E3 ubiquitin ligase Parkin (Figure 2; Kondapalli et al., 2012; Ordureau et al., 2014). In turn, Parkin further ubiquitinates OMM proteins (Chan et al., 2011; Sarraf et al., 2013) inducing recruitment of autophagosomes and lysosomes (Matsuda et al., 2010). Mutations of both *PINK1* and *Parkin* (*PARK2*) are the most common cause of autosomal recessive PD with predominantly early onset (Table 2; Kilarski et al., 2012; Truban et al., 2017). Thus, impaired mitochondrial quality control is obviously a key factor contributing to the loss of dopaminergic neurons in PD. However, the extensive functions of PINK1 and Parkin are not fully elucidated. Loss of PINK1 activity has been primarily related to decreased mitochondrial membrane potential as well as complex I and III deficiency (Amo et al., 2014). Furthermore, mitochondrial Ca^2+^ overload-induced cell death of PINK1-deficient dopaminergic neurons suggests an involvement of PINK1 in Ca^2+^ handling (Kostic et al., 2015). Despite the conserved role of Parkin in diverse mammalian cell lines, its relevance in neuronal mitophagy has been questioned. Some studies reported an only moderate Parkin contribution to degradation of damaged mitochondria in primary neurons (Cai et al., 2012; Grenier et al., 2013), whereas others confirmed the importance of Parkin for mitophagy in these cells (Grenier et al., 2014) and especially axons (Ashrafi et al., 2014). Interestingly, Parkin-deficient mice (Perez and Palmiter 2005) as well as rats (Dave et al., 2014) did not reveal a PD-related phenotype, and neither did the absence of Parkin influence selective neurodegeneration in MitoPark mice (Sterky et al., 2011). Accordingly, alternative mitophagy key players have been recently identified to regulate mitochondrial turnover in absence of Parkin (Villa et al., 2018). However, Parkin depletion caused degeneration of SNc dopaminergic neurons and motor symptoms upon impaired mtDNA replication (Pickrell et al., 2015), highlighting the importance of Parkin for dopaminergic neuron survival in face of accumulating mtDNA deletions.

Mitophagy depends on specific receptors which interact with the autophagosomal protein LC3 (microtubule-associated protein 1A/1B light chain 3). Among them, p62 was found to act as receptor for degradation of ubiquitinated proteins (Johansen and Lamark 2011). p62 affinity to ubiquitinated cargo is thereby facilitated by the kinases ULK1 (Unc-51 like autophagy activating kinase), CK2 (casein kinase 2) and TBK1 (TANK binding kinase 1) (Deng et al., 2017). Mutations in p62 encoding *SQSTM1* have been identified in patients suffering from ALS (Figure 2) associated with frontotemporal dementia (Rubino et al., 2012; Le Ber et al., 2013; Yilmaz et al., 2020). *In vitro* studies revealed that mutant *SQSTM1* is unable to recognize LC3, limiting p62 recruitment to autophagosomes (Goode et al., 2016; Nozaki et al., 2021). According to this, p62-positive aggregates have been detected in the spinal cord of ALS patients (Mizuno et al., 2006; Sasaki 2011) as well as ALS-related animal models (Sasaki and Iwata, 2007; Kato 2008; Hadano et al., 2010). Regarding the large number of distinct proteins that is found to aggregate in ALS, it is likely that motor neuron degeneration underlies impaired selective autophagy. Besides its interaction with LC3, p62 is further involved in the anti-oxidative stress response pathway. *SQSTM1* mutation exacerbated TDP-43 dependent stress response due to impaired p62 interaction with KEAP1 (Kelch-like ECH-associated protein 1) (Deng et al., 2020) and gives reason to suggest a pathological dual mode of action of mutated *SQSTM1*. Recently, homozygous mutations in *SQSTM1* have been linked to childhood-onset cerebellar ataxia (Haack et al., 2016; Vedartham et al., 2019). Skin fibroblasts from patients revealed a decreased autophagic flux with concurrently reduced mitochondrial OXPHOS activity. Downregulation of *SQSTM1* further led to atrophy in the cerebellum following axonal degeneration in a zebrafish model (Muto et al., 2018), indicating cerebellar susceptibility to disturbed p62 function.

Nevertheless, p62 rather functions as a general autophagic receptor for ubiquitinated proteins and has been further linked to the ubiquitin proteasome system (Liu et al., 2016; Nam et al., 2017). Thus, p62 is thought to play a secondary role in mitophagy, while as primary mitophagic receptors, OPTN and NDP52 have been identified (Wong and Holzbaur 2014; Heo et al., 2015; Lazarou et al., 2015; Richter et al., 2016). It is suggested that those are recruited to mitochondria *via* their ubiquitin binding domain, which then activates a positive feedback loop in order to recruit autophagosomes (Padman et al., 2019). Mutations of *OPTN* as well as *TBK1*, encoding a kinase which contains OPTN as a substrate, are found in patients with ALS (Figure 2; Wong and Holzbaur, 2014; Cirulli et al., 2015; Freischmidt et al., 2015; Feng et al., 2019). In particular, mutant *OPTN* failed to associate with the mitochondrial surface (Wong and Holzbaur, 2014; Lazarou et al., 2015), while mutant *TBK1* reduced recruitment of OPTN and LC3 to damaged mitochondria (Moore and Holzbaur, 2016). Loss of function of either OPTN or TBK1 impaired mitophagy resulting in accumulation of defective mitochondria (Evans and Holzbaur, 2019). Taken together, these data suggest impaired mitochondrial quality control *via* autophagy to be a critical factor for mitochondrial dysfunction found in ALS. However, the role of disturbed mitophagy in sporadic cases still needs to be elucidated.

Another pathway for mitochondrial quality control has been recently described involving so called mitochondrial-derived vesicles (MDVs) (Figure 2), which shuttle mitochondrial cargo to peroxisomes or lysosomes (Sugiura et al., 2014). Here, selected proteins can be degraded instead of the entire organelle (McLelland et al., 2014), which renders MDV-derived mitochondrial removal more selective than mitophagy. Formation of MDVs is promoted by the retromer complex, which was initially described in endosome-to-Golgi as well as endosome-to-plasma membrane transport (Seaman, 2012). Since VPS35 is part of the retromer complex and mutations in the encoding gene are associated with autosomal dominant PD (Vilarino-Guell et al., 2011; Zimprich et al., 2011), VPS35 is thought to play an important role in mitochondrial quality control. Indeed, VPS35 has been shown to associate with mitochondrial membranes and mediates vesicle transport between mitochondria and peroxisomes (Braschi et al., 2010). VPS35 has also been linked to mitochondrial fusion and fission, since mutant *VPS35* caused mitochondrial fragmentation, explained by decreased MFN2 levels in response to mitochondrial E3 ubiquitin ligase 1 (MUL1) degradation (Tang et al., 2015), and increased turnover of Drp1 (Wang et al., 2016). A recent study further showed reduced mitochondrial clearance in dopaminergic neurons derived from patients carrying the *VPS35* mutation p.D620N (Hanss et al., 2020), providing first evidence for impaired mitophagy by mutant *VPS35* in PD.

*In vitro* analysis of induced pluripotent stem cell-derived motor neurons from CMT2A patients, revealed upregulation of PINK1 and Parkin together with an increased autophagic flux (Rizzo et al., 2016). The authors therefore suggest that low mitochondrial mass upon *MFN2* mutation is rather induced by enhanced mitophagy instead of impaired mitochondrial biogenesis. Indeed, MFN2 has been shown to be further involved in OMM tethering to ER membranes in order to limit mitochondrial ubiquitination and delay mitophagy (McLelland and Fon 2018; McLelland et al., 2018). Mutant *MFN2* in CMT2A could hence induce mitophagy by its displacement from contact sites between mitochondria and ER. Simultaneously, decreased protein levels of MFN2 detected in various neurodegenerative diseases could consequently reflect enhanced mitophagy as a consequence of expanding mitochondrial dysfunction. Besides *MFN2*, Charcot-Marie-Tooth disease can be further caused by mutations in *GDAP1* (Zimon et al., 2011). A recent study showed that the OMM protein GDAP1 links mitochondria and lysosomes by binding LAMP1 (lysosomal associated membrane protein 1), while loss of GDPA1 affects basal autophagy (Figure 2) (Cantarero et al., 2020). These data provide new evidence for altered mitochondrial quality control contributing to the pathogenesis of peripheral neuropathies.

Besides gene mutations that are directly linked to mitochondrial removal pathways, accumulation of dysfunctional mitochondria and misfolded or mutant proteins, such as Aβ, Tau, a-Syn, TDP-43 and HTT, point to impaired autophagy in idiopathic cases as well (Monaco and Fraldi 2020). In brains of AD patients, autophagy-related protein Beclin 1 was decreased, which was linked to decreased autophagic flux and higher Aβ accumulation (Pickford et al., 2008). In addition, Parkin-dependent mitophagy was highly induced, while protein levels of Parkin decreased during disease progression (Ye et al., 2015), suggesting disturbed recognition of defective mitochondria at later stages of AD. It is likely, that pTau further interferes with mitophagy by disrupting axonal transport (Combs et al., 2019) and thereby retrograde movement of defective mitochondria. In PD, impaired mitophagy is known to be a shared feature of both idiopathic and familial PD (Hsieh et al., 2016). Interestingly, α-Syn aggregates have been recently shown to cause upregulation of Miro1 leading to delayed mitochondrial removal (Shaltouki et al., 2018). Since HTT physiologically interacts with p62 and the ULK1 complex (Ochaba et al., 2014; Rui et al., 2015), it could be speculated that in HD, mHTT might be unable to provide the proposed scaffold function and thereby affects mitophagy.

### Calcium Handling

**FIGURE 3:**
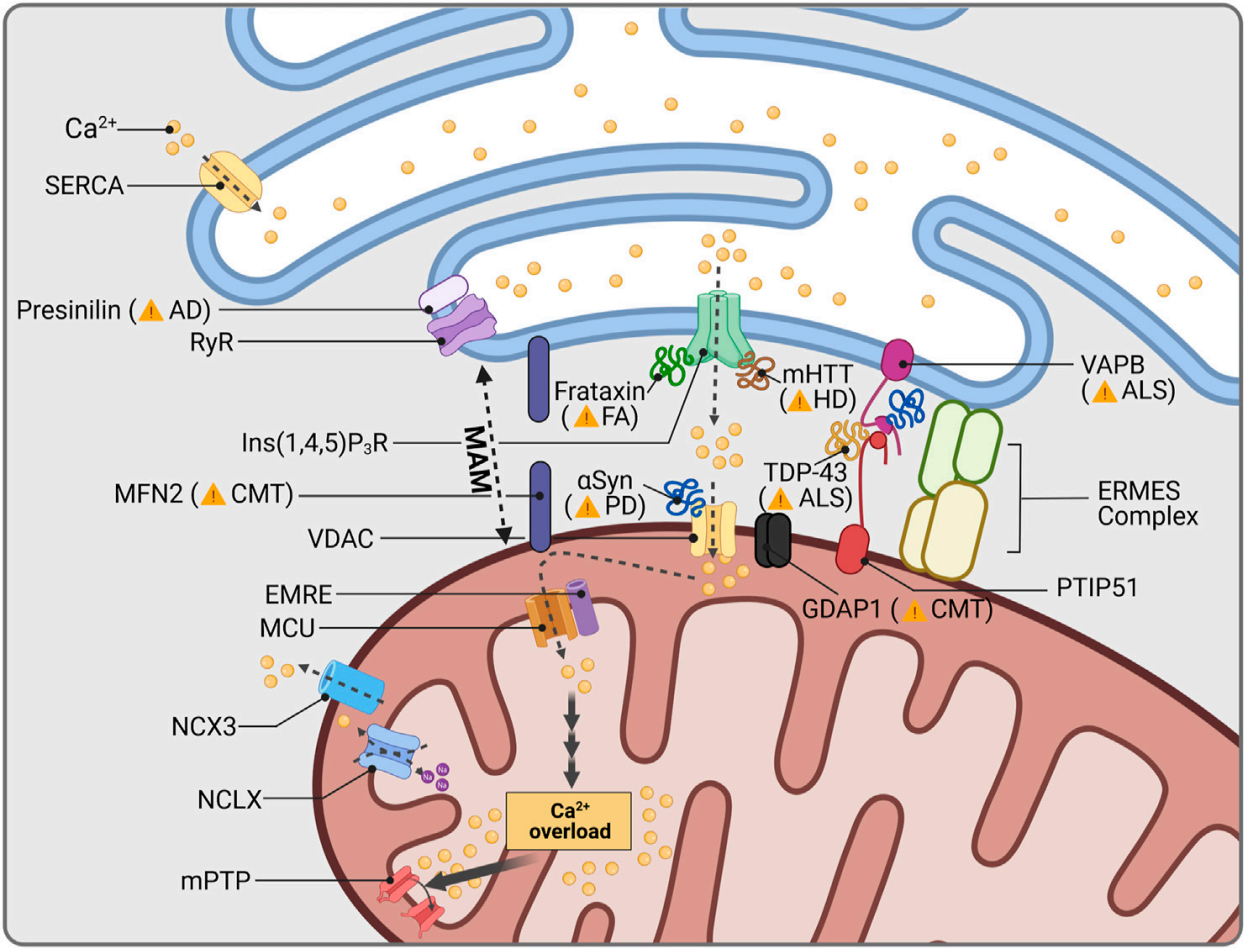
Mitochondrial Ca^2+^ and neurodegeneration. The ER is the main cellular organelle to store Ca^2+^. ER Ca^2+^ stores are released into the cytoplasm through Ryanodine Receptor channels (RyR), whose activity is regulated by the AD-related protein Presenilin. Cellular Ca^2+^ homeostasis is centralized in mitochondria-associated membranes (MAMs), the interphase between mitochondria and the ER. MAM structure is stabilized by a group of proteins, among them CMT2A-related MFN2, as well as ALS-linked VAPB and PTIP51. The OMM protein GDAP1 is further present in MAMs and is linked to impaired Ca^2+^ signaling, depending on the *GDAP1* mutation causing CMT. Other proteins which are associated with human diseases are known to interact with proteins resident in MAMs: Friedreich Ataxia (FA) protein frataxin, stabilizes MAMs through control of ROS levels; aggregates of mHTT increase Ca^2+^ release through Ins(1,4,5)P3R; mutations in the ALS-related protein TDP-43 disrupt interaction between VAPB and PTIP51; and PD related αSyn has been found to increase mitochondrial Ca^2+^ permeability through VDAC interaction. Mitochondrial Ca^2+^ overload induces opening of the mitochondrial permeability transition pore (mPTP), cytochrome c release and apoptosis activation.

Mitochondria serve as mobile regulators of local and bulk intracellular Ca^2+^ concentrations. Upon mitochondrial uptake, Ca^2+^ boosts mitochondrial metabolism and OXPHOS (McCormack and Denton 1979; Jouaville et al., 1999), and can thereby modulate the cell’s performance (Rizzuto et al., 2012). In neurons, mitochondria further impact on electrophysiological activity by shaping somato-dendritic as well as axonal and presynaptic Ca^2+^ oscillations (Billups and Forsythe, 2002; Kwon et al., 2016; Marland et al., 2016; de Juan-Sanz et al., 2017; Vaccaro et al., 2017). Under basal conditions, Ca^2+^ concentrations inside mitochondria resemble those measured in the cytoplasm (100–200 nM), however, mitochondria are able to buffer at 10–20-fold higher concentrations if needed. Pore-forming voltage-dependent anion-selective channel proteins (VDACs) mediate Ca^2+^ uptake from the cytosol or ER contact sites into the mitochondrial intermembrane space, where Ca^2+^ is forwarded into the mitochondrial matrix *via* the mitochondrial Ca^2+^ uniporter (MCU) complex (Figure 3; Giorgi et al., 2018). Ca^2+^ thereby follows the negative mitochondrial inner membrane potential (-150 to -180 mV) built up by the respiratory chain (Friel and Tsien 1994; Babcock et al., 1997). Efflux of one Ca^2+^ from the mitochondrial matrix occurs in exchange of three to four Na^+^ through the mitochondrial Na^+^/Ca^2+^ antiporter (Jung et al., 1995; Dash and Beard 2008). From the intermembrane space, Ca^2+^ extrudes into the cytosol *via* VDACs and the Na^+^/Ca^2+^ exchanger 3, respectively (Scorziello et al., 2013; Giorgi et al., 2018).

Since excessive amounts of cytosolic Ca^2+^ have been linked to excitotoxicity (Sattler and Tymianski 2000; Hardingham and Bading 2003), and mitochondrial Ca^2+^ overload has been shown to trigger cell death through opening of the mitochondrial permeability transition pore (Giorgi et al., 2012; Marchi et al., 2018), more and more attention was paid to imbalanced mitochondrial Ca^2+^ handling as putative cause of neurodegeneration in common and rare disorders. Neuronal Ca^2+^ oscillations promote pacemaker activity and fuel mitochondrial OXPHOS in SNc dopaminergic neurons (Surmeier et al., 2010). However, Ca^2+^ oscillations are a disservice to these vulnerable neuron population, as they cause mitochondrial oxidant stress by at that time unknown mechanisms (Guzman et al., 2010). Simultaneously, SNc dopaminergic neurons with low expression of the Ca^2+^-binding protein calbindin were found to be more affected in *post mortem* brains of PD patients (Damier et al., 1999) as well as in PD-related animal models (German et al., 1992; Dopeso-Reyes et al., 2014), indicating a potential neuroprotective effect of calbindin. The metabolic challenge following increased Ca^2+^ levels together with low intrinsic Ca^2+^ buffer capacities, is thought to render SNc dopaminergic neurons especially vulnerable to mitochondrial dysfunction (Philippart et al., 2016). Correspondingly, various voltage-gated Ca^2+^ channels have been associated with the selective loss of SNc dopaminergic neurons in PD, including L-type (Chan et al., 2007; Guzman et al., 2010; Surmeier et al., 2012; Duda et al., 2016), T-type (Guzman et al., 2018; Tabata et al., 2018) and R-type channels (Benkert et al., 2019). In line with this, we could recently show that upon respiratory chain deficiency, SNc dopaminergic neurons perish due to high Ca^2+^ loads, which finally impair the mitochondrial antioxidant defense (Ricke et al., 2020).

The increased levels of cytosolic Ca^2+^ in SNc dopaminergic neurons have been further linked to the expression of α-Syn, suggesting a converging pathway for these two pathogenic factors in PD (Lieberman et al., 2017). In support of this, a recent study showed that a-Syn dynamically binds to VDACs and modifies their Ca^2+^ permeability (Figure 3; Rosencrans et al., 2021). It is therefore likely that pathological aggregations of α-Syn might affect VDAC binding and thus mitochondrial Ca^2+^ buffering. In addition, wild type α-Syn is physiologically localized to mitochondria-ER contact sites called mitochondria-associated ER membranes (MAM) (Poston et al., 2013) which mediates the transfer of Ca^2+^ (Cali et al., 2012). However, mutant α-Syn dissociates from MAM causing mitochondrial fragmentation and decreased contacts between mitochondria and the ER (Table 3; Guardia-Laguarta et al., 2014). Accordingly, mutant α-Syn disturbs Ca^2+^ exchange between mitochondria and ER through disruption of the integral ER protein VAPB (vesicle-associated membrane protein-associated protein B) binding (Paillusson et al., 2017), showing mitochondrial Ca^2+^ homeostasis to be potentially affected by pathogenic α-Syn variations in PD patients. Recently, MAM have been shown to further depend on MIRO1: Heterozygous mutations in the gene encoding MIRO1 (*RHOT1*) were identified in two PD patients, while fibroblasts from these patients presented a decrease in MAM and impaired Ca^2+^ homeostasis (Grossmann et al., 2019). These findings were subsequently approved in patient-derived neurons (Berenguer-Escuder et al., 2020) and confirm the importance of mitochondrial Ca^2+^ handling for SNc dopaminergic neuron survival.

**TABLE 3:** Proteins related to disturbed mitochondrial Ca^2+^ handling.

Protein	Associated disease	Result of malfunction	References
Aβ	Alzheimer’s disease (AD)	Enhanced Ca^2+^ transfer from ER to mitochondria	Ferreiro et al. (2008), Calvo-Rodriguez et al. (2019)
AFG3L2	Spinocerebellar Ataxia	Accumulation of MCU-EMRE complexes (↑ mitochondrial Ca^2+^ overload)	Konig et al. (2016)
α-Syn	Parkinson’s disease (PD)	Dissociation from MAMs, decreased contacts between mitochondria and ER	Guardia-Laguarta et al. (2014)
Frataxin	Friedreich’s Ataxia	Disturbed Ca^2+^ homeostasis	Rodriguez et al. (2020)
GDAP1	Charcot-Marie-Tooth disease type 4A (CMT4A)	Mitochondrial mislocation at SOCE (↓mitochondrial Ca^2+^ uptake)	Pla-Martín et al. (2013), Gonzalez-Sanchez et al. (2017)
		Decreased contacts between mitochondria and ER	Cantarero et al. (2020)
HTT	Huntington’s disease (HD)	Enhanced activation of Ins(1,4,5)P_3_R causing Ca^2+^ release from ER stores	Tang et al. (2003), Tang et al. (2005)
MFN2	Charcot-Marie-Tooth disease type 2A (CMT2A)	Decreased MAM formation (↓mitochondrial Ca^2+^ uptake)	de Brito and Scorrano (2008), Gbel et al. (2020)
MICU1	Spinocerebellar Ataxia	Accumulation of MCU-EMRE complexes (↑ mitochondrial Ca^2+^ overload)	Tsai et al. (2016)
MIRO1	Parkinson’s disease (PD)	Decreased contacts between mitochondria and ER, impaired Ca^2+^ homeostasis	Grossmann et al. (2019), Berenguer-Escuder et al. (2020)
Presenilin	Alzheimer’s disease (AD)	Increased cellular and mitochondrial Ca^2+^ levels	Sanz-Blasco et al. (2008), Calvo-Rodriguez et al. (2016)
TDP-43	Amyotrophic Lateral Sclerosis (ALS)	Decreased contacts between mitochondria and ER (↓mitochondrial Ca^2+^ uptake)	Stoica et al. (2016)
VAPB	Amyotrophic Lateral Sclerosis (ALS)	Disturbed Ca^2+^ homeostasis	Nishimura et al. (2004), Morotz et al. (2012)

Disturbed Ca^2+^ homeostasis has been identified early as a characteristic feature in motor neurons of ALS patients (Siklos et al., 1996) and could be mimicked by various *in vitro* and *in vivo* models expressing mutant *SOD1* (Carri et al., 1997; Siklos et al., 1998; Damiano et al., 2006), *TDP-43* (Stoica et al., 2014) and *FUS* (Stoica et al., 2016), respectively. Interestingly, recovery of physiological Ca^2+^ concentrations in motor neurons was delayed upon AMPA (*α*-amino-5-methyl-3-hydroxisoxazolone-4-propionate) receptor activation (Guatteo et al., 2007). Since AMPA receptors are highly expressed at postsynaptic terminals and, similar to SNc dopaminergic neurons, they only possess poor intrinsic Ca^2+^ buffering capacities, motor neurons in ALS are supposed to perish by Ca^2+^-induced excitotoxicity (Van Den Bosch et al., 2000; Grosskreutz et al., 2010). Motor neurons therefore especially rely on proper Ca^2+^ buffering by mitochondria. Accordingly, disruption of MAM has been reported in several ALS-related models (Stoica et al., 2014; Bernard-Marissal et al., 2015; Stoica et al., 2016). VAPB (vesicle associated membrane protein associated protein B and C) interacts with the mitochondrial protein PTPIP51 (protein tyrosine phosphatase-interacting protein 51) in order to regulate Ca^2+^ exchange (De Vos et al., 2012). Mutations in *VAPB* have been linked to familial ALS (Figure 3; Nishimura et al., 2004) showing disturbed Ca^2+^ homeostasis (Morotz et al., 2012). Moreover, TDP-43 was shown to disrupt the VAPB-PTPIP52 pathway (Stoica et al., 2016), suggesting a similar mechanism in idiopathic ALS cases with pathogenic TDP-43 accumulation. In support of this, expression levels of *VAPB* were found to be reduced in the spinal cord of idiopathic ALS patients (Anagnostou et al., 2010). Recent analysis of motor neurons derived from patients carrying mutations in *TDP-43* revealed that Ca^2+^-permeable AMPA and NMDA receptor upregulation was associated with reduced mitochondrial Ca^2+^ uptake due to an imbalance between MICU1 and MICU2 (Dafinca et al., 2020). Thus, it is likely that in ALS, glutamate excitotoxicity might generally underlie defective mitochondrial Ca^2+^ buffering, induced by MCU complex imbalance or MAM disruption. However, a recent study indicated mutation-specific alterations in Ca^2+^ dynamics of patient-derived motor neurons, which would consequently require differential treatment strategies (Bursch et al., 2019).

In line with its contribution to impaired mitochondrial Ca^2+^ handling in ALS, disrupted MAM are likely to be involved in motor neuron loss of CMT2A patients (Bernard-Marissal et al., 2019). Besides the scaffolding multisubunit protein complex ERMES (ER-mitochondria encounter structure) (Kornmann et al., 2009), MFN2 is also involved in the connection of mitochondria and ER (McLelland et al., 2018; McLelland et al., 2018). Since silencing or depletion of MFN2 leads to decreased MAM formation associated with impaired mitochondrial Ca^2+^ uptake (Table 3; de Brito and Scorrano 2008; Gbel et al., 2020), perturbations of mitochondrial Ca^2+^ handling might play a crucial role for motor neuron survival in Charcot-Marie-Tooth disease as well. In accordance to this, GDAP1 has been linked to the so called store-operated Ca^2+^ entry (SOCE). In general, SOCE is activated following depletion of ER Ca^2+^ stores, which is controlled by the ER transmembrane protein STIM1 (stromal interaction protein 1). Low Ca^2+^ levels within the ER induce STIM1 dimerization and its diffusion to plasma membrane contact sites where it directly binds TRPC channels, in particular ORAI1, activating Ca^2+^ entry into the cytosol (Liu et al., 2015). These membrane junctions are stabilized by proteins known as junctophilins which also serve as signaling hubs for the process (Sahu et al., 2019). In this context, mitochondria function as local Ca^2+^ buffer and avoid early inhibition of ORAI1 channels, thereby allowing restoring proper Ca^2+^ levels in the ER. GDAP1 deficiency has been shown to cause mitochondrial mislocation at SOCE, avoiding proper Ca^2+^ buffering and inducing the inhibition of ORAI1 (Pla-Martin et al., 2013; Gonzalez-Sanchez et al., 2017). This feature is associated with progressive decline of motor neurons in mice (Barneo-Munoz et al., 2015) and ER stress (Civera-Tregon et al., 2021). It should be noted that missense mutations associated with Charcot-Marie-Tooth disease have different effects in SOCE activity. While dominant inherited missense mutation generate incremented SOCE, recessive inherited mutations lead to a complete loss of function and inhibition of Ca^2+^ entry (Gonzalez-Sanchez et al., 2017). Noteworthy, mutations in *JPH1*, encoding Junctophilin-1, were found to increase the severity of a dominant inherited *GDAP1* mutation (Pla-Martin et al., 2015).

Multiple types of spinocerebellar ataxia have been found to be associated with alterations of neuronal Ca^2+^ levels (Patron et al., 2018). The importance of controlled mitochondrial Ca^2+^ handling, in particular, is evidenced by MICU1-deficient mice, presenting ataxia following degeneration of Purkinje cell projections (Liu et al., 2016). MICU1 depletion leads to mitochondrial Ca^2+^ overload through accumulation of MCU-EMRE (essential MCU regulator) complexes, allowing unrestricted Ca^2+^ influx into the mitochondrial matrix (Table 3; Tsai et al., 2016). Moreover, MCU-EMRE complexes accumulate upon depletion of AFG3L2 and again disrupt gatekeeping of the MCU complex (Konig et al., 2016). Lowering Purkinje cell Ca^2+^ levels in *AFG3L2* haploinsufficient mice by decreasing glutamate stimulation, in turn, could rescue Purkinje cell degeneration and the ataxic phenotype (Maltecca et al., 2015). This indicates a beneficial effect of reducing cytosolic Ca^2+^ concentrations on Purkinje cells of patients carrying *AFG3L2* mutations and, simultaneously, points to glutamate-dependent excitotoxicity as cause of neuron death. However, preliminary data by Langer and colleagues showed that *MCU* deletion in Purkinje cells lacking AFG3L2 did not influence neurodegeneration (Patron et al., 2018), suggesting that modifying mitochondrial Ca^2+^ levels alone is not sufficient to prevent degeneration of Purkinje cells in AFG3L2-related spinocerebellar ataxia. Nevertheless, it remains to be seen how MCU depletion is truly affecting mitochondrial Ca^2+^ levels and especially cytosolic Ca^2+^ concentrations. Accordingly, decreasing cytosolic Ca^2+^ loads as well as glutamate transmission in Purkinje cells might be the treatment of choice in order to prevent degeneration in spinocerebellar ataxia. Impaired Ca^2+^ handling might thereby be a general pathological factor underlying ataxias. In support of this, frataxin, whose encoding gene is mutated in Friedreich’s ataxia, has been recently shown to be localized to MAMs and to stabilize them through protection against ROS (Rodriguez et al., 2020). Modulation of Ca^2+^ levels could further rescue the axonal decline of frataxin-deficient sensory neurons *in vitro* (Molla et al., 2017).

In HD, abnormalities of mitochondrial Ca^2+^ handling have been first detected in isolated mitochondria from affected individuals. While having reduced Ca^2+^ buffering capacities, mitochondria from HD patients further showed mitochondrial membrane depolarization at low Ca^2+^ concentrations in contrast to those from control subjects (Panov et al., 2002). In accordance to this, investigation of striatal cells from HD-related models revealed that mitochondria were able to buffer low Ca^2+^ levels, but failed at higher concentrations (Lim et al., 2008; Quintanilla et al., 2013). The failure in mitochondrial buffering of high Ca^2+^ loads has been subsequently linked to chronic Ca^2+^ leakage from the ER: in medium spiny neurons, mHTT enhanced activation of the intracellular Ca^2+^ release channel inositol(1,4,5)-triphosphate receptor (Figure 3; Tang et al., 2003) causing Ca^2+^ release from ER stores through ryanodine receptors (Tang et al., 2005). Inhibition as well as stabilization of ryanodine receptors correspondingly attenuated Ca^2+^ leak and cell death (Suzuki et al., 2012), suggesting intracellular Ca^2+^ store depletion through mHTT to be the causative event for neuronal Ca^2+^ overload, and impaired mitochondrial Ca^2+^ handling to be a consequence (Table 3). Furthermore, reduced Ca^2+^ uptake by mitochondria would exacerbate Ca^2+^-induced cytotoxicity. However, increased mitochondrial Ca^2+^ influx has been reported in HD-related models as well (Oliveira et al., 2007; Wang J.-Q et al., 2013; Pellman et al., 2015). Inhibition of the MCU complex thereby prevented mitochondrial permeability transition pore opening and hence degeneration of medium spiny neurons *in vitro* (Tang et al., 2005). Regarding the effects of mHTT on mitochondrial dynamics, it could be speculated that MAM formation is additionally influenced and would further affect neuronal Ca^2+^ buffering. It is therefore likely that it needs to target both the ER and mitochondria in order to correct cytosolic Ca^2+^ levels and prevent Ca^2+^-induced loss of medium spiny neurons in HD.

Disturbed cytosolic Ca^2+^ homeostasis had been primarily linked to AD pathogenesis by familial cases associated with mutations of Presenilin 1 (*PS1*) and 2 (*PS2*) (Table 3; LaFerla 2002). These mutations cause high accumulation of Aβ aggregates, which have been shown to trigger cell death following increased intracellular (Busche et al., 2008; Kuchibhotla et al., 2008) as well as mitochondrial Ca^2+^ levels (Sanz-Blasco et al., 2008; Calvo-Rodriguez et al., 2016). Presenilins are directly involved in cytosolic Ca^2+^ regulation by interaction with ryanodine receptors (Chan et al., 2000) and their modulatory protein sorcin, respectively (Pack-Chung et al., 2000). Moreover, they have been shown to be involved in ER-mitochondria coupling (Filadi et al., 2016), suggesting mutant forms to disrupt neuronal Ca^2+^ handling by altering ER-mediated Ca^2+^ storage and MAM formation. In fact, increased contacts between mitochondria and the ER have been found in patient-derived fibroblasts, human brain tissue and AD-related animal models (Hedskog et al., 2013; Area-Gomez and Schon 2017). High MAM number could be detrimental to mitochondria and trigger cell death, especially with regard to the ability of Aβ aggregates to additionally induce Ca^2+^ transfer from the ER to mitochondria (Ferreiro et al., 2008; Calvo-Rodriguez et al., 2019). Noteworthy, increased ER-mitochondria contacts (Perreault et al., 2009) as well as elevated mitochondrial Ca^2+^ levels have been linked to AD models showing Tau pathology as well (Quintanilla et al., 2009; Britti et al., 2020). Since Aβ aggregates and pTau are cellular hallmarks for idiopathic AD, limiting mitochondrial Ca^2+^ uptake could be of general importance in order to preserve mitochondrial functionality and to prevent Ca^2+^-induced neurodegeneration. In line with this, a recent study revealed that Aβ-induced mitochondrial Ca^2+^ overload was MCU-dependent and preceded neurodegeneration in mice, indicating putative pharmacological intervention by blocking MCU (Calvo-Rodriguez et al., 2020).

## Selective Vulnerability

It is striking that despite the fundamental importance of mitochondria for neurons, specific neuronal populations perish in the disorders presented above. Especially considering familial cases, in which proteins carrying monogenic mutations are widespread in both the central and peripheral nervous system, cell-type specific factors are likely to contribute to the selective vulnerability (Table 4). Concerning the distinct mitochondrial processes being reported to be affected in the diseases mentioned above, cell-intrinsic properties might enhance the risk of neuron death induced by mitochondrial defects.

**TABLE 4:** Most affected neuron populations in selected neurological disorders.

Cell-type	Intrinsic factors linked to vulnerability	Associated disease
SNc dopaminergic neurons	Unmyelinated, long and extremely branched axons	Parkinson’s disease (PD)
	High density of axonal mitochondria	
	High energetic demands	
	Dopamine metabolism	
	K-ATP channel dysregulation	
	Ca^2+^ oscillations by voltage-gated Ca^2+^ channels low intrinsic Ca^2+^ buffering capacity	
Spinal motor neurons	Extraordinary large axons	Amyotrophic Lateral Sclerosis (ALS), Peripheral Neuropathies (Charcot-Marie-Tooth disease)
	Large soma and complex dendritc trees	
	Marge motor unit size (one axon suppling up to 2,000 muscle fibres) innervation of fast-twitch, fast-fatigable muscle fibres	
	Low intrinsic Ca^2+^ buffering capacity	
Purkinje cells	Giant dendritic trees	Spinocerebellar Ataxia, Spastic Ataxia, Friedreich’s Ataxia
	Strong dependence on intracellular Ca^2+^ homeostasis	
GABAergic medium spiny neurons	Strong dependence on BDNF	Huntington’s disease (HD)
	High excitability	
	Large glutamatergic input from cortical neurons	
Pyramidal neurons in the Entorhinal Cortex Layer II (ECII) and the hippocampal CA1 region	Early accumulation of neurofibrillary tangles	Alzheimer’s disease (AD)
	Strong dependence on tau homeostasis	
	Glutamatergic neurotransmission	
	Low intrinsic Ca^2+^ buffering capacity	

### Substantia Nigra Pars Compacta Dopaminergic Neurons

In PD, different neuronal populations are affected in a variety of brain areas, with perished non-dopaminergic neurons being primarily linked to early non-motor symptoms, including anxiety and depression (Postuma et al., 2012). However, characteristic motor symptoms are caused by the loss of dopaminergic neurons in the SNc and the accompanying depletion of dopamine in the dorsal striatum (Michel et al., 2016; Obeso et al., 2017). An important factor for the neurons’ vulnerability is their neurotransmitter. Dopamine metabolism is associated with the production of various intermediate metabolites and reactive oxygen species (ROS), which impede antioxidant processes and can induce oxidative stress (Delcambre et al., 2016), a pathophysiological cellular state which has been early linked to PD pathogenesis (Jenner and Olanow 1996; Alam et al., 1997). Moreover, dopamine metabolism drives the accumulation of mtDNA deletions (Neuhaus et al., 2014; Neuhaus et al., 2017). Nevertheless, dopamine alone cannot be the reason for neurodegeneration in the SNc, since dopaminergic neurons in the neighboring ventral tegmental area (VTA) are much less affected in PD (Alberico et al., 2015). Electrophysiological characterization of these two dopaminergic midbrain populations thereby greatly contributed to a better understanding of potential pathological pathways. In contrast to the VTA (Khaliq and Bean 2010), SNc dopaminergic neurons in the SNc have to face high Ca^2+^ loads, resulting from activity-dependent Ca^2+^ oscillations and simultaneously low intrinsic Ca^2+^ buffering capacities, which have been associated with their selective neurodegeneration, as mentioned above. In addition, activation of mitochondrial OXPHOS by Ca^2+^ increases generation of ROS as a byproduct (Guzman et al., 2010), which might further promote the formation of mtDNA alterations (Lawless et al., 2020). Furthermore, Ca^2+^ is involved in the aggregation of αSyn (Dufty et al., 2007; Caraveo et al., 2014; Diepenbroek et al., 2014; Rcom-H'cheo-Gauthier et al., 2014), providing a direct link between enhanced Ca^2+^ levels and αSyn pathology in SNc dopaminergic neurons. Apart from voltage-gated Ca^2+^ channels, ATP-sensitive potassium (K-ATP) channels have been linked to SNc dopaminergic neuron death. Whereas protecting the cell from overexcitability and excitotoxicity under physiological conditions by mediating membrane hyperpolarization following metabolic demand (Liss and Roeper 2001), K-ATP channel opening in SNc dopaminergic neurons led to complete loss of electrophysiological activity and promoted cell death in a toxin-based model for PD. Noteworthy, this pathological mechanism was absent in dopaminergic neurons located in the VTA (Liss et al., 2005).

Another critical factor contributing to the vulnerability of SNc dopaminergic neurons is their morphology. Contrary to the VTA or the olfactory bulb, dopaminergic neurons within the SNc possess unusually long and extremely branched axons which are connected to a large number of nerve cells (Matsuda et al., 2009; Pacelli et al., 2015). A single axon of a human SNc dopaminergic neuron is thereby estimated to have a total length of 4.5 m and give rise to up to 2.4 million synapses (Bolam and Pissadaki 2012). This unique architecture of concurrently unmyelinated axon terminals (Braak et al., 2004) is an extreme challenge to mitochondrial bioenergetics and is accompanied by a high density of axonal mitochondria (Pacelli et al., 2015).

### Motor Neurons

Motor neuron degeneration is a pathological hallmark of ALS as well as several peripheral neuropathies, including CMT2A. Whether upper (corticospinal) or lower somatic motor neurons, both populations possess extraordinary long axons in order to enable neurotransmission from the primary motor cortex, down the spinal cord to the most distant muscle fibers of our body (Stifani 2014). This renders them critically dependent on proper axonal transport, and especially on mitochondrial trafficking in order to preserve a functional mitochondrial pool in far distance to the cell body. Concerning ALS, however, neurodegeneration seems to be rather complex, since not all motor neurons equally perish. Those neurons within the oculomotor (CNIII), trochlear (CNIV) and abducens (CNVI) nuclei are more or less resistant to neurodegeneration in ALS, allowing patients to maintain communication *via* eye movements (Kubota et al., 2000; Caligari et al., 2013) when speech problems are too severe. Whereas the majority of skeletal muscles reveal a single contact between each muscle fiber and incoming axon (*en plaque*), extraocular muscles can be innervated by oculomotor neurons in a grape-like structure (*en grappe*) (Zimmermann et al., 2013). In addition, oculomotor neurons possess small somas as well as dendritic trees (Ulfhake and Cullheim 1981; Highstein et al., 1982), and only innervate up to five muscle fibers (Enoka 1995). Spinal motor neurons, in contrast, innervate at least 300 muscle fibers, in large muscles such as the medial gastrocnemius supplying up to 2,000 muscle fibers (Burke et al., 1971; Burke and Tsairis 1973). Single fiber innervation and large motor unit size are accompanied with high energetic demands in order to preserve neurotransmission, and hence muscle contraction.

Morphological differences are also invoked concerning the selective vulnerability among spinal motor neurons in ALS: early degenerating motor neurons innervate a large number of fast-twitch, fast-fatigable, glycolytic muscle fibers, which are responsible for high-force movements. Spinal motor neurons which supply slow-twitch, fatigue-resistant muscle fibers, however, are more finely structured and rather robust against cell death (Nijssen et al., 2017). Interestingly, these motor neurons are even temporarily able to compensate the early loss of those which innervate fast-twitch, fast-fatigable muscle fibers (Frey et al., 2000; Schaefer et al., 2005). The robustness of surviving motor neurons in ALS is further related to their high intrinsic Ca^2+^ buffering capacity (Alexianu et al., 1994), highlighting again the importance of proper neuronal Ca^2+^ handling especially for susceptible spinal motor neurons innervating fast-twitch muscles.

### Purkinje Cells

The cerebellum is essential for the execution of goal-directed movement and coordination of body and limb posture. As they provide the sole output of the cerebellar cortex, Purkinje cells are considered to be the functionally most important cell type of this brain region. These large, pear-shaped neurons receive inputs from excitatory climbing fibers as well as parallel fibers originating from cerebellar granule cells and the inferior olivary nucleus within the brainstem, respectively. With their unbranched axons, Purkinje cells project to cerebellar nuclei located in the white matter, such as the dentate gyrus, and regulate neuronal output from the cerebellum to the brainstem and thalamus by GABAergic transmission (Cerminara et al., 2015). Purkinje cells are primarily characterized by their giant dendritic trees, which enable up to 150,000 synaptic connections per cell (Kandel et al., 2000). In addition, they are able to generate two types of action potential trains: high rate (30–100 Hz) simple spiking upon spontaneous activity or activation by parallel fibers (Armstrong and Rawson 1979), as well as low rate (∼1 Hz) complex spiking following activation by climbing fibers (Eccles et al., 1966). Histological detection of Purkinje cells is enabled by labeling of calbindin, which is abundantly expressed. Loss of calbindin in Purkinje cells caused motor coordination deficits associated with altered time course and amplitude of fast Ca^2+^ transients (Barski et al., 2003), highlighting the importance of intracellular Ca^2+^ buffering for Purkinje cell function. Cell-type specific genetic profiling recently revealed a variety of genes to be uniquely present in Purkinje cells. Among them, there were several genes associated with the development of autosomal dominant spinocerebellar ataxia. Interestingly, all of them are involved in intracellular Ca^2+^ homeostasis by either controlling mitochondrial Ca^2+^ uptake (*AFG3L2*), Ca^2+^ release from ER stores (*ITPR1*), glutamate receptor-mediated Ca^2+^ signaling (*TRPC3*), and fast Ca^2+^ entry within dendritic spines (*CACNA1G*) (Huang and Verbeek 2019), highlighting the strong dependence on intracellular Ca^2+^ homeostasis for Purkinje cell survival as well.

### Medium Spiny Neurons

While being located in the striatum, GABAergic medium spiny neurons present the major input region of the basal ganglia. Upon dopaminergic input by the SNc, medium spiny neurons either facilitate (direct pathway) or suppress movement (indirect pathway), depending on whether they express dopamine D1 or D2 receptors. Activation of D1 medium spiny neurons additionally leads to inhibition of the D2 population, and vice versa, according to the current basal ganglia model (McGregor and Nelson 2019). Although neurodegeneration in HD involves diverse brain regions, including the cerebral cortex, thalamus and hypothalamic nuclei, medium spiny neurons within the striatum are most severely affected and responsible for the symptomatic motor defects (Halliday et al., 1998; Vonsattel 2008). Until today, the special vulnerability of these striatal neurons is not fully understood. It is known that HD is caused by the CAG repeat expansion in the *HTT* gene (MacDonald et al., 1993), however, mHTT is expressed throughout the CNS and expression levels are neither enhanced in the striatum nor in cortical neurons projecting to the striatum (Trottier et al., 1995). One hypothesis for the special vulnerability of the striatum to mHTT involves its strong dependence on brain-derived neurotrophic factor (BDNF). Besides its critical importance during brain development, BDNF is required for long-term survival of adult medium spiny neurons and involved in the regulation of both dendritic morphology and spine number (Baydyuk and Xu 2014). Protein levels of BDNF were found to be reduced in the striatum of HD patients (Ferrer et al., 2000) as well as HD-related animal models expressing *mHTT* (Apostol et al., 2008; Gharami et al., 2008), which has been linked to interference of *mHTT* with synthesis and transport of BDNF (Zuccato et al., 2001; Gauthier et al., 2004). Interestingly, striatal neurons depend on BDNF supply through SNc dopaminergic neurons and particularly cortical projection neurons (Altar et al., 1997; Baquet et al., 2004), indicating early loss of cortico-striatal connectivity in HD (Novak et al., 2015) to additionally contribute to lack of striatal BDNF.

Another reason for the striatal vulnerability in HD may be provided by the action of Rhes, a small GTPase which is highly enriched in the striatum. This protein has been shown to interact with mHTT and then promote a toxic effect by causing ubiquitin-like modification (Subramaniam et al., 2009). Correspondingly, Rhes depletion in a HD mouse model improved striatal atrophy and motor symptoms (Swarnkar et al., 2015). A recent study showed that Rhes can form tunneling nanotubes *in vitro* and thereby enabled transfer of mHTT between striatal cells (Sharma and Subramaniam 2019), persuading the authors to suggest a similar mechanism *in vivo*. On the other side, the expression of Rhes in medium spiny neurons has not been clearly defined, and other brain regions, which are not affected in HD, have been shown to express it as well.

Interestingly, medium spiny neurons of the indirect pathway are found to degenerate earlier than those involved in the direct pathway. This is in line with the symptomatic course of disease: chorea is part of early HD stages, resulting from missing movement suppression, whereas akinesia and dystonia occur later in disease due to decreased movement initiation (Raymond et al., 2011). One possible explanation for the early death of indirect pathway medium spiny neurons could be the exclusive expression of the BDNF receptor TrkB, which might render those neurons more vulnerable to the lack of BDNF (Baydyuk et al., 2011). Another factor could be the differential innervation. Although both striatal populations are innervated by cortical projection neurons, medium spiny neurons of the indirect pathway receive excitatory inputs from so called pyramidal tract cortical neurons, which is accompanied with larger glutamate release (Ballion et al., 2008). In addition, cortical circuits are thought to be even more active in early HD stages preceding motor symptoms (Burgold et al., 2019) and indirect pathway neurons have been shown to be more excitable than direct pathway neurons (Gertler et al., 2008). Thus, it is likely that medium spiny neurons of the indirect pathway degenerate early due to an enhanced risk for glutamate-mediated excitotoxicity and simultaneously increased energetic demands.

### Pyramidal Neurons in the Entorhinal Cortex Layer II and Hippocampal Cornu Ammonis 1 Region

Cognitive decline and memory loss in AD are caused by atrophy of the entorhinal cortex, hippocampus as well as neocortical areas. This atrophy is linked to the accumulation of both Aβ aggregates and neurofibrillary tangles. Whereas Aβ plaques are relatively widespread in the AD brain (Thal et al., 2002), tau pathology reveals the same distribution pattern as neurodegeneration (Braak and Braak 1991) and correlates with clinical symptoms (Brier et al., 2016). In particular, pyramidal neurons of ECII and the hippocampal CA1 region have been shown to selectively perish in early stages of the disease. Other neuron populations, however, such as pyramidal neurons of CA2, CA3, the primary visual cortex, primary somatosensory cortex, and granule cells of the dentate gyrus, are spared from degeneration (Hyman et al., 1984; Arnold et al., 1991; Gomez-Isla et al., 1996; Fukutani et al., 2000; Bussiere et al., 2003). Neurons within the ECII reveal relatively high energetic demands (Hevner and Wong-Riley 1992), indicating an enhanced susceptibility to additional stressors. The same might be true for CA1 pyramidal neurons, which present high sensitivity toward decreased glucose and oxygen delivery (Montagne et al., 2016). In addition, both ECII and CA1 pyramidal neurons are glutamatergic and hence susceptible to NMDA-mediated excitotoxicity, which could be further provoked by Aβ aggregates (Wang and Reddy 2017). Inhibitory interneurons of the neocortex expressing high levels of Ca^2+^-binding proteins, in contrast, are less affected (Hof et al., 1993; Fu et al., 2017), suggesting intracellular Ca^2+^ homeostasis to be an important factor for neuron survival in AD.

Interestingly, the spread of Aβ and tau pathology in the brains of AD patients could be recapitulated by clustering of genes whose proteins are likely to co-aggregate with Aβ plaques and neurofibrillary tangles in healthy individuals. Vulnerable brain regions revealed high expression of aggregation-promoting factors, including chaperones and post-translational modifiers (Freer et al., 2016). This suggests that even vulnerable neuron populations have a genetic predisposition to early accumulate Aβ aggregations and neurofibrillary tangles. However, this study did not provide cell-type specific information. Comparison of gene expression profiles between excitatory and inhibitory neurons identified a gene whose protein is involved in the regulation of pathological tau accumulation. Exclusively expressed in inhibitory neurons, BAG3 (BCL2-associated athanogene 3) was consequently linked to the vulnerability of excitatory neurons in AD (Fu et al., 2019). Eventually, a recent study investigating the molecular profile of vulnerable and resistant neurons in AD placed microtubule dynamics at the center of pathogenesis. In particular, a regulator of tau splicing was identified to be dysregulated in vulnerable ECII pyramidal neurons and might explain initial formation of neurofibrillary tangles (Roussarie et al., 2020), which then spread to other brain regions *via* hippocampal CA1 neurons (Kaufman et al., 2018).

## Discussion

It is out of question that mitochondrial defects are involved in neurodegeneration of many common and rare neurodegenerative diseases. However, before considering to target mitochondria, in order to design novel therapeutic strategies against these disorders, it needs to be clarified how much the disrupted mitochondrial pathways eventually contribute to neuron death. Are they crucial factors or do they rather arise secondary to other pathological processes? The development of neurodegenerative diseases is unequivocally linked to aging. During aging, the activity of cellular quality control mechanisms decreases (Rubinsztein et al., 2011) while ROS amounts rise (Stefanatos and Sanz 2018), which should result in a stronger presence of fragmented and defective mitochondria. Interestingly, mitochondrial fragmentation is often observed in both common and rare diseases, with neurons frequently showing altered levels of mitochondrial dynamics proteins. While disrupted mitochondrial fusion or enhanced fission could hence be seen as a disease-spanning feature, mitochondrial fragments could also result from decelerated removal of defective mitochondria in the first place. The presence of fragmented mitochondria observed in neurological disorders must thus be interpreted with caution, since it might reflect a phenomenon related to aging. Impairment of mitochondrial dynamics, mtDNA maintenance or Ca^2+^ handling, however, could cause additional susceptibility to specific neuron populations, which ultimately tips the balance from survival toward death.

### Huntington’s Disease

In HD, mitochondrial defects, such as impaired trafficking and Ca^2+^ handling, are primarily caused by mHTT. Cell-intrinsic properties of GABAergic medium spiny neurons do not indicate extraordinary vulnerability to mitochondrial dysfunction. In fact, the strong dependence on BDNF signaling rather sustains the special susceptibility of these neurons to mHTT. Instead of facilitating mitochondrial transport or modifying cytosolic Ca^2+^ levels, which could be beneficial to slow down neurodegeneration for a certain period of time, it is thus wiser to focus on the removal of mutant HTT in order to prevent neuron death in the long term. In this context, a promising new treatment strategy based on antisense oligonucleotide injection *via* lumbar puncture is currently tested and has already been shown to reduce mHTT levels by 40–60% (Lempriere 2019).

### Alzheimer’s Disease

Similar to HD, it is likely that mitochondrial dysfunction plays rather a secondary role for neurodegeneration in AD and arises as a consequence of Aβ and pTau pathology. A probably primary contribution of neurofibrillary tangles and maybe also Aβ aggregates is confirmed by cell-intrinsic properties of ECII and CA1 pyramidal neurons, showing high dependence on tau homeostasis. Correspondingly, reducing Aβ aggregates and pTau levels in the brain is still in the focus of AD research. The antisense oligonucleotide technique has been applied recently and could achieve a reduction of both the amyloid precursor protein (Chang et al., 2018) and pTau (DeVos et al., 2017) in preclinical trials. However, some studies still suggest that mitochondrial defects are upstream of Aβ and pTau pathology, fueling the “mitochondrial cascade hypothesis,” which puts mitochondrial dysfunction at the heart of AD development (Swerdlow et al., 2014). Mitochondrial function has been shown to be impaired even before the accumulation of Aβ deposits (Yao et al., 2009; Leuner et al., 2012; Mao et al., 2012) and mitochondrial impairment *via* toxins or genetic ablation of ROS suppressing proteins accelerated Aβ pathology (Esposito et al., 2006; Chen et al., 2015). Formation of Aβ aggregates was accordingly facilitated by ROS upon mitochondrial dysfunction (Gwon et al., 2012). Interestingly, complex I inhibition *via* rotenone treatment in rats leads to increased pTau in neurons, astrocytes and oligodendrocytes (Hoglinger et al., 2005). Furthermore, the loss of mitochondrial proteins, such as the protease AFG3L2 (Kondadi et al., 2014) and the ROS scavenger SOD2 (Melov et al., 2007), is associated with pTau presence, indicating that mitochondrial dysfunction promotes tauopathy in the brain. In line with this, inhibition of mitochondrial fragmentation (Wang et al., 2017) and upregulation of PGC-1α, which induces mitochondrial biogenesis (Katsouri et al., 2016), could ameliorate Aβ pathology and cognitive defects in mice. Recently, by using a series of *in vitro* and *in vivo* models, stimulation of mitophagy was shown to reduce Aβ deposits as well as pTau formation, and hence reversed cognitive impairment (Fang et al., 2019). Thus, future approaches to treat AD might be designed in such a way that Aβ and pTau levels in the brain are lowered, and that mitochondrial function is simultaneously improved, e.g., by enhancing mitochondrial quality control. In addition, mitochondrial-targeted reagents suppressing the formation of ROS could be beneficial, as indicated by treatment with tetra-peptide SS31 (Reddy et al., 2017) or Mito-Ubiquinone (MitoQ), which improves cognitive decline in triple transgenic AD mice (McManus et al., 2011) and is currently tested in early onset AD patients (NCT03514875).

### Peripheral Neuropathies

In peripheral neuropathies, the mitochondrial impact on selective neurodegeneration might be more extensive. Due to their extraordinary long axons, it is quite comprehensible that motor neurons are especially vulnerable to disruption of mitochondrial dynamics and especially trafficking of mitochondria, as seen in Charcot-Marie-Tooth disease caused by mutations in MFN2, GDAP1 as well as KIF1B, encoding the mitochondrial motor KIF1Bβ (Zhao et al., 2001). Besides mitochondrial fission and fusion, mutations in *MFN2* and *GDAP1* further affect mitophagy and mitochondrial Ca^2+^ handling mediated *via* MAMs. In these cases, disrupted mitochondrial dynamics are clearly the causative event for motor neuron loss. Treatments of peripheral neuropathies might consequently involve genetic approaches to introduce wild type MFNs and GDAP1, as shown recently in a *MFN2* transgenic mouse model (Zhou et al., 2019), or pharmacological approaches to target these proteins. In fact, agonists for MFN2 have been identified and shown to rescue mitochondrial fusion as well as trafficking *in vitro* (Rocha et al., 2018) and could therefore present a promising therapeutic tool for the future, especially for treatment of CMT2A.

### Amyotrophic Lateral Sclerosis

For ALS, it is rather difficult to postulate a primary cause of mitochondrial dysfunction for motor neuron death because of the disease’s complex degenerative pattern, the variety of distinct gene mutations, and the presence of aggregate-prone proteins. Whether of primary cause or not, mitochondrial fragmentation at least participates in ALS linked to SOD1 mutation, since inhibition of mitochondrial fission by overexpression of dominant negative *Drp1 K38A* normalized morphology and transport of mitochondria, leading to increased motor neuron survival (Song et al., 2013). However, in familial cases showing mutations in *SQSTM1*, *OPTN* or *TBK1*, mitochondrial fragmentation might be rather secondary to impaired mitophagy in the first place, resulting in the accumulation of shortened mitochondria. Stimulating mitophagy might hence be a promising target to prevent mitochondrial dysfunction in both familial and sporadic ALS. Simultaneously, for familial cases harboring mutations in *SOD1*, *TDP*-43 and *FUS*, as well as for sporadic cases revealing characteristic protein aggregations, mitochondrial defects likely result from misfolded proteins. In these cases, stimulation of bulk autophagy should address both pathways by promoting mitochondrial turnover and removal of such aggregate-prone proteins. Accordingly, within the last years, different autophagy modulators, including rapamycin, trehalose or rilmenidine, have been tested. However, they provided mixed results depending on the model used, administration route, and off-target effects (Madruga et al., 2021), highlighting both the complexity and heterogeneity of this disease. Finally, targeting cytosolic Ca^2+^ levels might be an additional promising approach, considering the low Ca^2+^ buffering capacity and the high risk of glutamate induced excitotoxicity for vulnerable spinal motor neurons innervating fast-twitch muscles. Noteworthy, future therapies would further need to include neuromuscular junctions, since prevention of motor neuron loss *per se* might not recover the lost axonal connections to muscle fibers, once they have retracted (Gould et al., 2006; Dewil et al., 2007). Focusing only on the maintenance of spinal motor neurons might therefore not improve clinical symptoms at later stages of ALS.

### Spinocerebellar Ataxias

In several types of spinocerebellar ataxia fragmented mitochondria are observed in Purkinje cells. However, mitochondrial fragmentation is likely not the primary cause of selective neurodegeneration, but occurs as a side effect of upstream malfunctions, as indicated e.g. in case of *AFG3L2* mutations. In addition, shuttling of mitochondria throughout the enormous dendritic tree of Purkinje cells highly depends on prior mitochondrial fission in order to be able to leave the soma (Delettre et al., 2000; Ishihara et al., 2009), with defective fission leading to shortening of the dendritic arbor (Fukumitsu et al., 2016). This renders Purkinje cells rather vulnerable to a pathologically hyperfused mitochondrial network. Consequently, targeting mitochondrial fission could be a potential treatment of patients suffering from spinocerebellar ataxia type 7, which has been linked to mitochondrial elongation (Ward et al., 2019). Furthermore, the enhanced susceptibility to mtDNA alterations following impaired replication highlights a strong dependence of Purkinje cells on mitochondrial OXPHOS, which might underlie the high energetic demand due to the special neuron morphology or an especially high turnover of respiratory chain complexes. Noteworthy, many spinocerebellar ataxias are linked to CAG nucleotide repeat expansions and the subsequent aggregation of toxic polyglutamine protein (Paulson et al., 2017). In these cases, reducing polyglutamine protein levels by using antisense oligonucleotides (Toonen et al., 2016) or small interfering RNA (Ramachandran et al., 2014) is probably of greater priority. After all, concerning the marked susceptibility of Purkinje cells to MCU impairment as well as the variety of monogenic mutations affecting Ca^2+^ homeostasis that have been linked to spinocerebellar ataxia, disrupted Ca^2+^ handling is likely to be a crucial factor for Purkinje cell loss. Potentially, future approaches aiming at successfully reducing cytosolic Ca^2+^ levels, ideally in distinct cell types, could be used to prevent Ca^2+^-induced cytotoxicity.

### Parkinson’s Disease

Mitochondrial dysfunction plays a crucial role for the selective neurodegeneration in PD, as evidenced by complex I inhibiting toxins causing parkinsonism (Langston et al., 1983; Betarbet et al., 2000), an especially high accumulation rate of mtDNA deletions in idiopathic cases, as well as the wide variety of monogenic mutations impairing mitochondrial function in familial forms. Remarkably, all mitochondrial processes mentioned above had been linked to the loss of SNc dopaminergic neurons. It is therefore not surprising that perished SNc dopaminergic neurons or parkinsonian features are further reported in cases of spinocerebellar ataxia (Palin et al., 2013; Tzoulis et al., 2013; Synofzik 2019) and CMT2A (Aerts et al., 2016), emphasizing their special vulnerability to mitochondrial defects. Cell type intrinsic properties are of critical importance for the high susceptibility of SNc dopaminergic neurons, which is highlighted by other dopamine neuron populations being spared upon mitochondrial dysfunction (Pass et al., 2020; Ricke et al., 2020). Thus, a lot of attention is currently paid on voltage-gated Ca^2+^ channels, since they present promising pharmacological targets to decrease the cytosolic Ca^2+^ burden and its associated pathology, including mitochondrial dysfunction as well as the formation of αSyn aggregates. The usage of dihydropyridines for instance, which block L-type channels, has been linked to a 20–30% lower risk for PD development in patients treated for high blood pressure (Lang et al., 2015). Despite controversial results regarding dose efficiency as well as specificity in mice, the dihydropyridine isradipine, has been tested in a phase III clinical trial on PD patients (Biglan et al., 2017). This clinical trial failed (Parkinson Study Group, S.-P. D. I. I. I. I., 2020), supporting recent concerns about the possible efficiency of the dosage which could be used (Ortner et al., 2017).

Mitochondrial-targeted reagents suppressing ROS, such as MitoQ, Mito-Apocynin or Szeto-Schiller peptide, reveal neuroprotective effects *in vitro* as well as in pharmacological-induced PD mouse models (Jin et al., 2014). Moreover, administration of Mito-Apocynin could attenuate motor defects and progressive degeneration of SNc dopaminergic neurons in MitoPark mice (Langley et al., 2017). However, a phase III clinical trial using MitoQ failed (Snow et al., 2010), which led to the oxidative stress hypothesis being fundamentally questioned.

Importantly, PD occurs in a very heterogeneous manner indicating a necessity for adjusted treatments for smaller patient cohorts instead of broadly-based therapy in the future. Considering PD cases presenting αSyn aggregates and mutant LRRK2, which are both likely to be upstream of mitochondrial defects, it appears necessary to decrease their presence in the brain by making use of antisense oligonucleotides or interfering RNA technologies. Noteworthy, not all PD cases reveal αSyn pathology, such as most of the patients harboring Parkin mutations, highlighting the chance for mitochondrial treatment approaches targeting mitochondrial trafficking or quality control.

## Conclusion

Despite the complex nature of neurodegenerative diseases, therapies targeting mitochondrial dysfunction represent a promising, potential tool to slow down or even stop neuron death. Depending on the mitochondrial impact on neurodegeneration, which varies from disease to disease, future treatments fueling mitochondrial function might be used either primarily or in a combinatory manner. Pharmacological compounds or therapies stimulating both mitochondrial biogenesis and turnover could therefore be used in addition to already established treatments, which mostly deal with clinical symptoms but not with the progressive loss of neurons. Convergent mitochondrial defects thereby reflect similar pathological pathways and hold the potential for disease-spanning treatment.
